# Salvianolic acid A alleviates H_2_O_2_-induced endothelial oxidative injury via miR-204-5p

**DOI:** 10.1038/s41598-024-62556-4

**Published:** 2024-05-24

**Authors:** Xilin Qiao, Shuyu Cao, Shuaiyu Chen, Yan Guo, Nipi Chen, Ying Zheng, Bo Jin

**Affiliations:** 1 School of Life Science, Zhejiang Chinese Medical University, Hangzhou, Zhejiang China; 2 Hangzhou TCM Hospital Affiliated to Zhejiang Chinese Medical University, Hangzhou, Zhejiang China; 3 The 903rd Hospital of the People’s Liberation Army, Hangzhou, Zhejiang China

**Keywords:** Cell death, Biochemistry, Molecular medicine

## Abstract

Oxidative stress induced endothelial dysfunction plays a particularly important role in promoting the development of cardiovascular diseases (CVDs). Salvianolic acid A (SalA) is a water-soluble component of traditional Chinese medicine *Salvia miltiorrhiza* Bunge with anti-oxidant potency. This study aims to explore the regulatory effect of SalA on oxidative injury using an in vitro model of H_2_O_2_-induced injury in human umbilical vein endothelial cells (HUVECs). In the study, we determined cell viability, the activities of Lactate dehydrogenase (LDH) and Superoxide dismutase (SOD), cell proliferation rate and intracellular reactive oxygen species (ROS). Flow cytometry was used to detect cell apoptosis. Western-blotting was used to evaluate the expression of cell senescence, apoptosis, autophagy and pyroptosis protein factors. The expression level of miRNA was determined by qRT-PCR. Compared with H_2_O_2_-induced HUVECs, SalA promoted cell viability and cell proliferation rate; decreased LDH and ROS levels; and increased SOD activity. SalA also significantly attenuated endothelial senescence, inhibited cell apoptosis, reversed the increase of LC3 II/I ratio and NLRP3 accumulation. Furthermore, miR-204-5p was regulated by SalA. Importantly, miR-204-5p inhibitor had similar effect to that of SalA on H_2_O_2_-induced HUVECs. Our results indicated that SalA could alleviate H_2_O_2_-induced oxidative injury by downregulating miR-204-5p in HUVECs.

## Introduction

Cardiovascular disease (CVD) is a leading cause of morbidity and mortality worldwide, especially among the aging population^1,2^. Endothelial dysfunction is an early marker of vascular damage linked to the disruption of the vascular homeostasis and represents a key step in the pathogenesis of CVDs. The redox imbalance is the main cause of endothelial dysfunction. Accumulating evidence suggested that oxidative stress occurs throughout the causes of endothelial cell (EC) dysfunction, leading to cellular senescence and death, including apoptosis, autophagic cell death, pyroptosis, necroptosis and ferroptosis^3–5^. It is crucial to rescue ECs from oxidative injury, implementation of effective interventions can alleviate endothelial dysfunction and offer important preventative and therapeutic benefits for patients with CVDs.

The senescence and death of ECs are not only adverse outcomes, but also causal contributors to endothelial dysfunction. The intracellular reactive oxygen species (ROS) when present in excess, result in oxidative stress, EC senescence and even cell death. However senescent ECs can produce high levels of ROS, mainly including superoxide, H_2_O_2_^6^. Then, H_2_O_2_ or ROS can induce multiple modes of EC death. Moreover, different modes of cell death may compensate for each other. EC autophagy is generally considered to be vasoprotective, which is attributed to its ability to balance the status of cellular redox and bioenergetics. Apoptosis is one of the most widely studied forms of programmed cell death, and excess ROS is a major driver of EC apoptosis^7^. Necroptosis, pyroptosis, and ferroptosis are three main modes of regulated necrosis, a majority of cell death events in EC dysfunction. Necroptotic inflammation can be enhanced by crosstalk with pyroptosis, NOD-like receptor family pyrin domain-containing 3 (NLRP3) inflammasome is considered as a key step of this crosstalk^8^. Herein, adequate protection of EC from oxidative stress mediated cellular senescence and death appears to be promising therapeutics.

Danshen, *Salvia miltiorrhiza* Bunge, is a traditional Chinese medicine widely used for the prevention and treatment of CVDs^9^. Salvianolic acid is the main active ingredients in *Salvia miltiorrhiza* Bunge, including Salvianolic acid A (SalA), Salvianolic acid B (SalB) and Salvianolic acid C and so on. The results of pharmacological researches have showed that Salvianolic acid exerts antioxidation, anti-inflammatory and antithrombotic effects^10^. In our previous work, we revealed that H_2_O_2_ could induce oxidative damage to human umbilical vein endothelial cells (HUVECs), which could be reversed by SalB^11^. However, the therapeutic mechanisms of SalA are not fully understood, and the regulation of signaling transduction pathways remains limited.

MicroRNAs (miRNAs) are a class of small non-coding RNAs that regulate gene expression post transcriptionally, and thus participate in various physiopathological processes, including cell proliferation, differentiation, cell death^12^. SalA was confirmed to protect blood-spinal cord barrier integrity by modulation of miRNA (miR-101a) expression in primary rat brain microvascular EC^13^. Therefore, miRNA could be a promising therapeutic target for SalA rescue endothelial oxidative injury. miR-204-5p is derived from the 5′ end arm of hsa-miR-204 precursors (pre-miRNAs)^14^. Growing evidence have proved that miR-204-5p plays crucial role in EC dysfunction^15,16^. Our previous works had showed that the expression of miR-204-5p was conspicuously elevated in H_2_O_2_ induced HUVECs^17^. Thus, we aimed to find whether SalA could rescue ECs from oxidative injury by targeting regulation of miR-204-5p.

## Results

### SalA ameliorated H_2_O_2_-induced HUVECs injury

**Figure 1:**
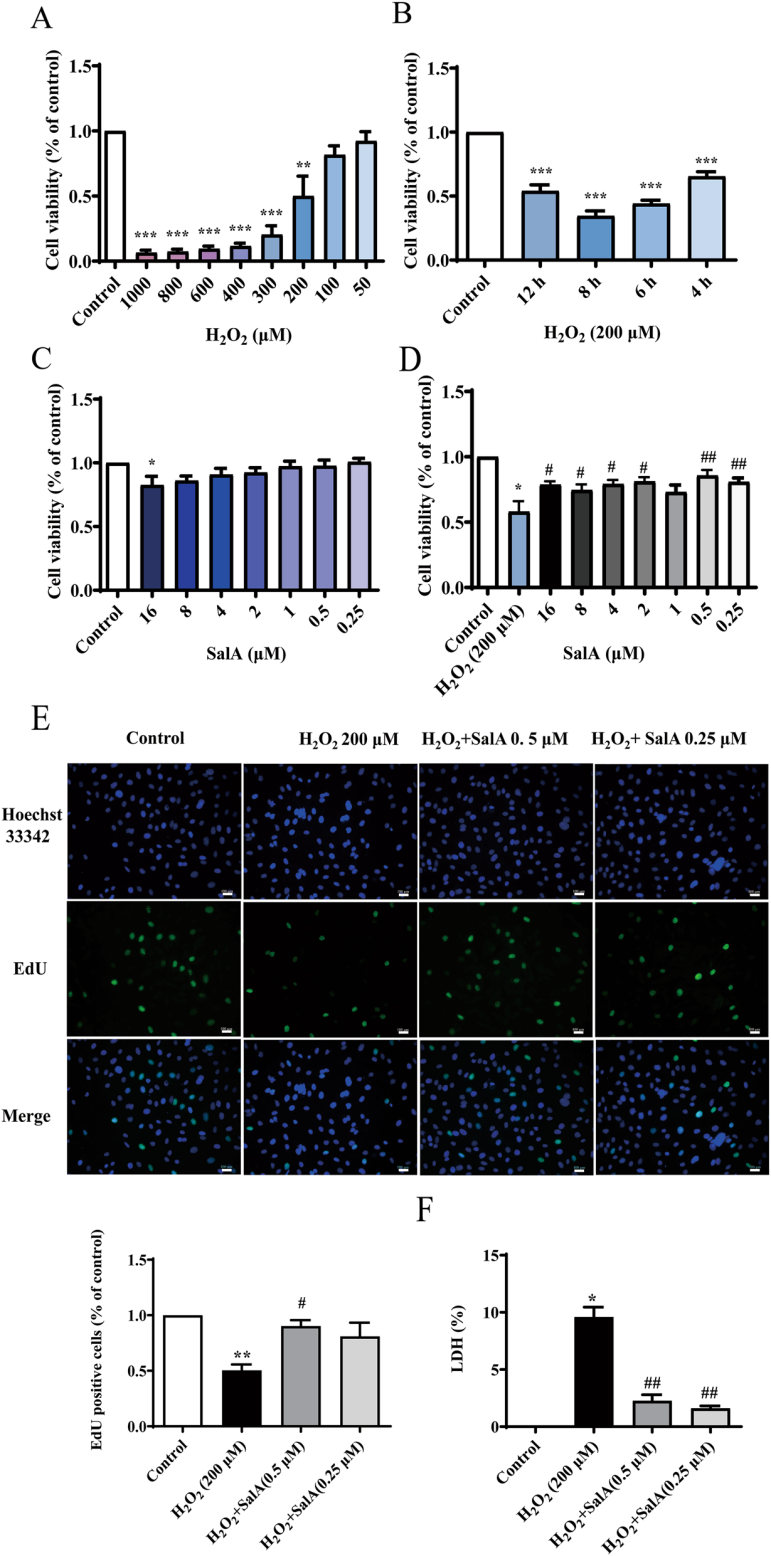
Effects of SalA on cell viability, cell proliferation and LDH release. (**A**) The effects of different concentration of H_2_O_2_ (50–1000 μM) treated for 4 h on the cell viability of HUVECs. (**B**) The effects of different treatment time of 200 μM H_2_O_2_ on the cell viability of HUVECs. (**C**–**F**) HUVECs were treated with SalA for 24 h after 12 h 200 μM H_2_O_2_ inducing: (**C**) The effect of SalA on the cell viability of HUVECs. HUVECs were treated with different concentration of SalA (0.25–16 μM). (**D**) The effect of SalA on the cell viability in H_2_O_2_-induced HUVECs. (**E**) The effect of SalA on the cell proliferation rate in H_2_O_2_-induced HUVECs were determined using a EdU assay. (**F**) The effect of SalA on LDH release in H_2_O_2_-induced HUVECs. Values are expressed as the mean ± SD (n = 3) (*P < 0.05, **P < 0.01, ***P < 0.001 vs. Control, ^#^*P* < 0.05, ^##^*P* < 0.01 vs. H_2_O_2_).

First, we used different concentrations of H_2_O_2_ to induce HUVECs for 4 h to construct oxidative damage models. The results of CCK-8 (Fig. 1A) showed that cell viability was inhibited to different degrees with increasing concentrations of H_2_O_2_ (50–1000 μM), which indicated that cell was damaged by oxidative stress. Among them, when the H_2_O_2_ concentration was 200 μM, the cell survival rate was around 50% and statistically different. Therefore, we chose 200 μM H_2_O_2_ to stimulate HUVECs in the subsequent experiments.

Then, we continued to optimal the H_2_O_2_ treatment time with the concentration of 200 μM. The results (Fig. 1B) showed that 200 μM H_2_O_2_ significantly inhibited the cell viability of HUVECs at 4 h, 6 h, 8 h,12 h treatment time. According to the recent reports and our results^18–20^, the treatment time of 12 h was selected to develop a model of oxidative damage.

To define the optimal concentration of SalA, a dose–response curve was performed within the previously established nontoxicity range from 0 to 16 μΜ, and examined for cell viability. SalA (0.25–8 μM) did not alter cell viability compared to the control group (Fig. 1C), but improved the cell viability compared to H_2_O_2_ group (*P* < 0.05), and SalA (0.25, 0.5 μM) significantly increased the cell viability against H_2_O_2_-induced injury (Fig. 1D) (*P* < 0.01). Therefore, we selected 0.25 μM and 0.5 μM as the optimal concentration of SalA in subsequent experiments.

The Edu assay was used to examine the effect of SalA treatment on cell proliferation. The results (Fig. 1E) showed that compared with the control group, H_2_O_2_ induced group was significantly decreased cell proliferation rate (*P* < 0.01). Compared with H_2_O_2_ group, SalA increased the cell proliferation rate, but only 0.5 μM SalA group exhibited a significant change (*P* < 0.05).

Lactate dehydrogenase (LDH) leakage as a marker for cell degradation was also monitored. LDH release in H_2_O_2_ group was highly increased than control group (*P* < 0.05). After treated with SalA (0.25, 0.5 μM), the LDH release rate was significantly inhibited (*P* < 0.01) (Fig. 1F).

Thus, SalA is effective in protecting HUVECs against cell injury caused by H_2_O_2_.

### SalA reduced oxidative stress in H_2_O_2_-induced HUVECs

**Figure 2:**
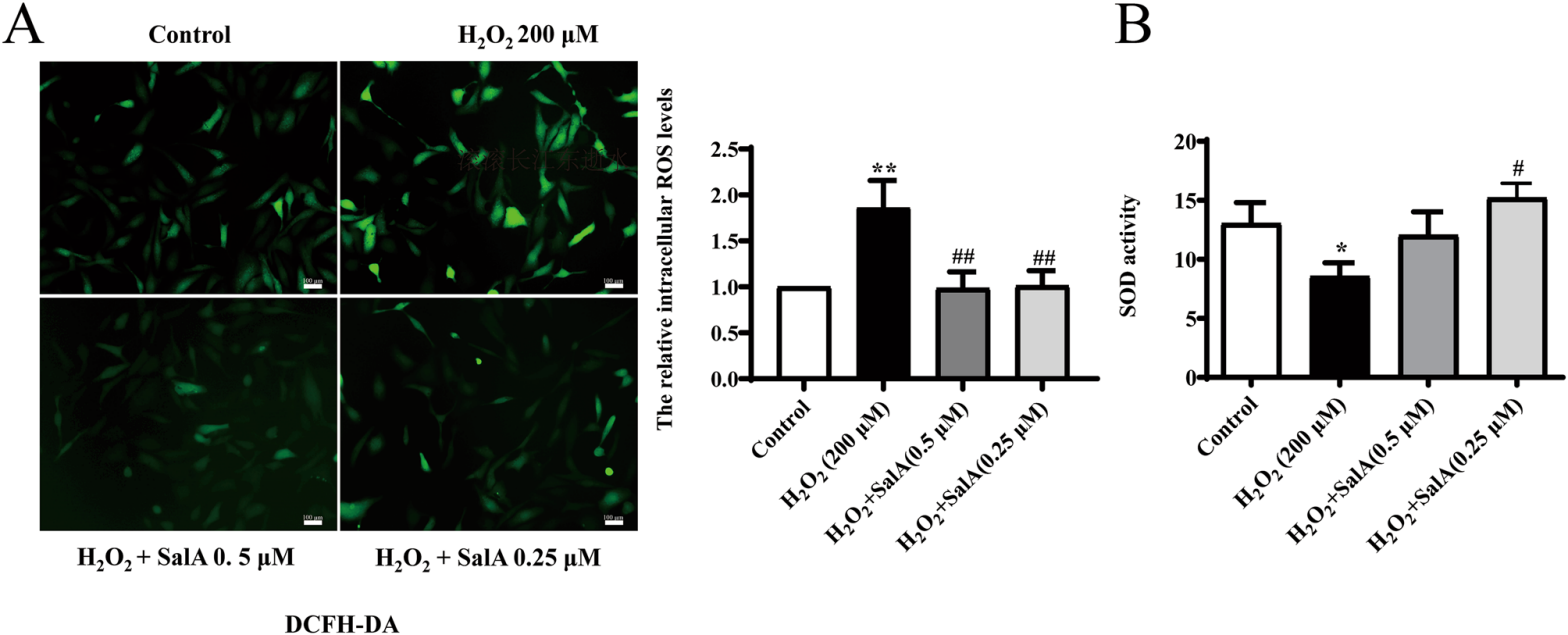
Effect of SalA on Intracellular ROS level and SOD activity in HUVECs induced by H_2_O_2_. HUVECs were treated with SalA for 24 h after 12 h 200 μM H_2_O_2_ inducing. (**A**) The effect of SalA on ROS level in H_2_O_2_-induced HUVECs were determined using a ROS assay. (**B**) The effect of SalA on SOD activity in H_2_O_2_-induced HUVECs. Values are expressed as the mean ± SD (n = 3) (*P < 0.05, **P < 0.01 vs. Control, ^#^*P* < 0.05, ^##^*P* < 0.01 vs. H_2_O_2_).

ROS is often used as an index to evaluate oxidation. Results as shown in Fig. 2A, ROS levels in H_2_O_2_ group was significantly higher than that in control group (*P* < 0.01). Compared with H_2_O_2_ group, SalA (0.25, 0.5 μM) treatment significantly decreased ROS levels in HUVECs (*P* < 0.01).

Superoxide dismutase (SOD) is one of the most important antioxidants. In Fig. 2B, the SOD activity in the H_2_O_2_ group was significantly lower than in the control group (*P* < 0.05), and SalA treatment ameliorated the decrease in SOD activity in H_2_O_2_ group, especially in 0.25 μM SalA group (*P* < 0.05).

### SalA attenuated cell senescence in H_2_O_2_-induced HUVECs.

**Figure 3:**
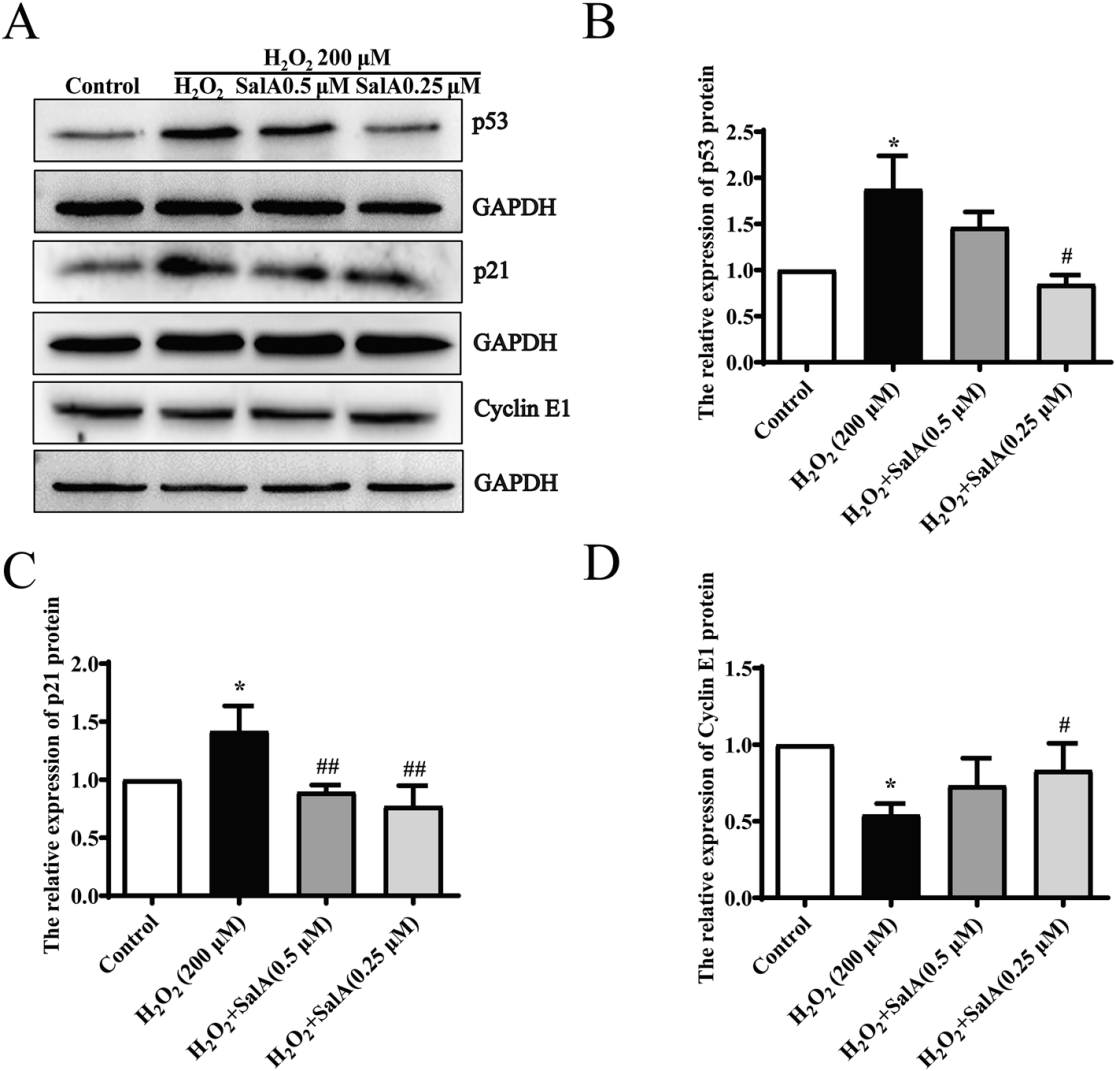
Effect of SalA on cell senescence in HUVECs induced by H_2_O_2_. HUVECs were treated with SalA for 24 h after 12 h 200 μM H_2_O_2_ inducing. (**A**) The effect of SalA on the (**B**) p53, (**C**) p21, (**D**) Cyclin E1, were detected by western blotting. Uncropped gel images are provided in the supplementary file. Values are expressed as the mean ± SD (n = 3) (*P < 0.05 vs. Control, ^*#*^*P* < 0.05, ^*##*^*P* < 0.01 vs. H_2_O_2_).

Oxidative stress can induce cell senescence, the expression levels of cell cycle-related proteins such as p53, p21, and Cyclin E1 were detected to analyze the cellular senescence with western blot analysis. In Fig. 3A–D, compared with the control group, our results showed that the expression levels of p53 and p21 were increased (*P* < 0.05) while Cyclin E1 expression level were decreased (*P* < 0.05) in H_2_O_2_ induced HUVECs. SalA treatment decreased the expression of p53 and p21 proteins, and increased Cyclin E1 level, in which 0.25 μM SalA had the most significant effect (*P* < 0.05, *P* < 0.01, *P* < 0.05).

### SalA attenuated programmed cell death in H_2_O_2_-induced HUVECs

**Figure 4:**
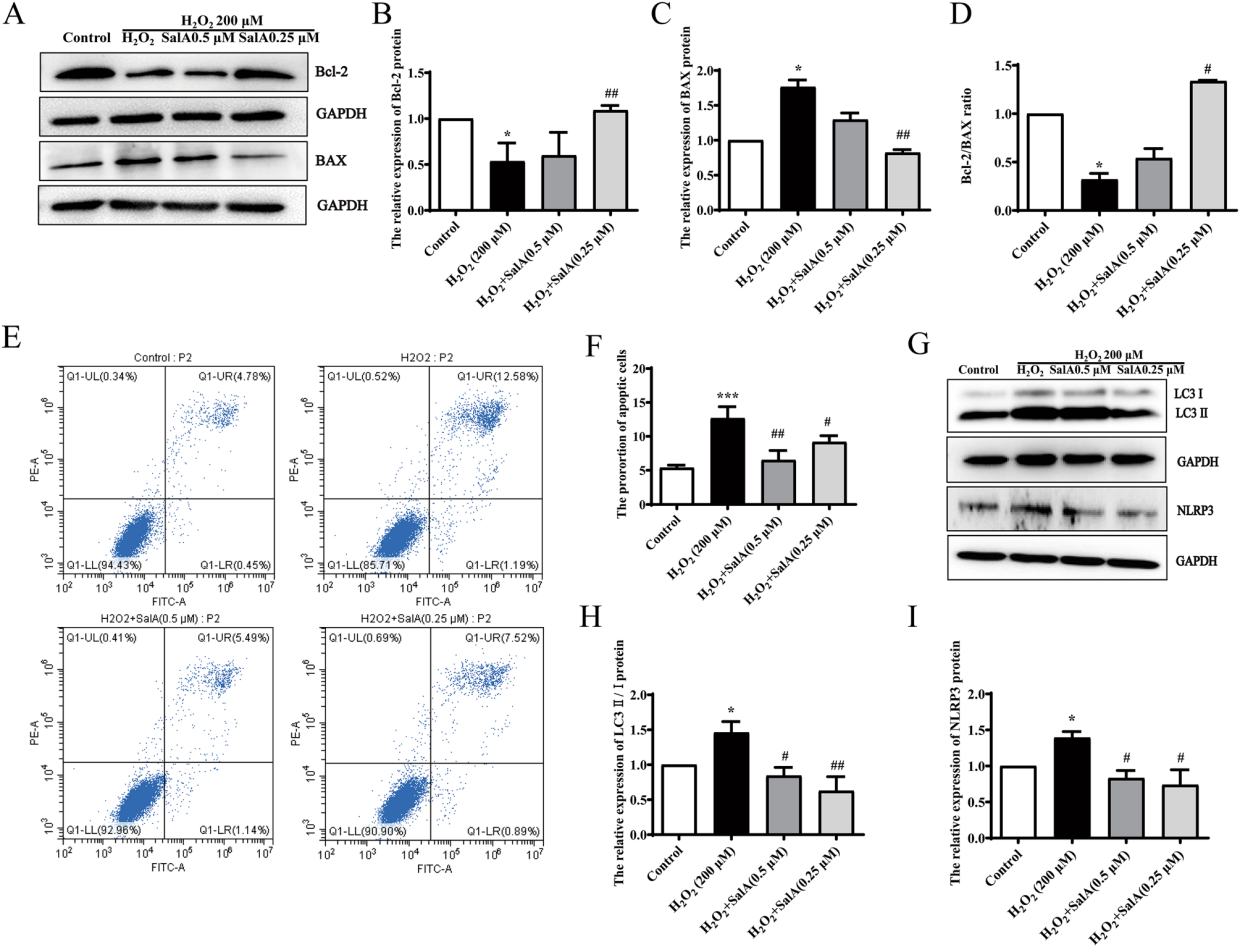
Effect of SalA on cell death in HUVECs induced by H_2_O_2_. HUVECs were treated with SalA for 24 h after 12 h 200 μM H_2_O_2_ inducing. (**A**) The effect of SalA on the protein expressions of (**B**) Bcl-2 and (**C**) BAX were detected by western blotting. (**D**) The expression ratio of Bcl-2/BAX. (**E**,**F**) Representative images of flow cytometry analysis of the effect of SalA on cell apoptosis rate. (**G**) The effect of SalA on the (**H**) LC3 II/I ratio and (**I**) NLRP3 protein expression were detected by western blotting. Uncropped gel images are provided in the supplementary file. Values are expressed as the mean ± SD (n = 3) (*P < 0.05, ***P < 0.001 vs. Control, ^*#*^*P* < 0.05, ^*##*^*P* < 0.01 vs. H_2_O_2_).

Apoptosis mostly led by oxidative stress, and Bcl-2 and BAX are the main regulatory genes of apoptosis. The ratio of Bcl-2/BAX can show the level of apoptosis. As shown in Fig. 4A–D, compared with control group, Bcl-2 protein expression diminished significantly (*P* < 0.05), and BAX protein increased substantially (*P* < 0.05) in H_2_O_2_ group. On the contrary, SalA treatment increased Bcl-2 level (*P* < 0.01) while reduced BAX level significantly (*P* < 0.01), and 0.25 μM SalA had significant effect by increasing the ratio of Bcl-2/BAX (*P* < 0.05). The cell flow result also indicated that SalA significantly reduced the apoptosis induced by H_2_O_2_ in Fig. 4E–F.

Autophagy is an evolutionarily conserved cellular process which is essential for cell survival and maintenance of intracellular homeostasis^21^. The level of LC3 II/I ratio was considered as a biomarker of the changes of autophagy in cells. The increase of LC3 II/I ratio in H_2_O_2_ group was significant (*P* < 0.05), whereas SalA (0.25, 0.5 μM) significantly inhibited LC3 II/I ratio in Fig. 4G–H.

NLRP3 is a key marker of inflammatory death induced by oxidative stress^22^. As result in Fig. 4I, compared with the control group, the protein expressions of NLRP3 were obviously upregulated (*P* < 0.05) in H_2_O_2_-induced HUVECs, while 0.25 μM and 0.5 μM SalA treatments reversed these aberrant changes (*P* < 0.05).

These findings indicated that SalA could attenuate H_2_O_2_-induced programmed cell death in HUVECs.

### SalA decreased miR-204-5p expression in H_2_O_2_-induced HUVECs.

**Figure 5:**
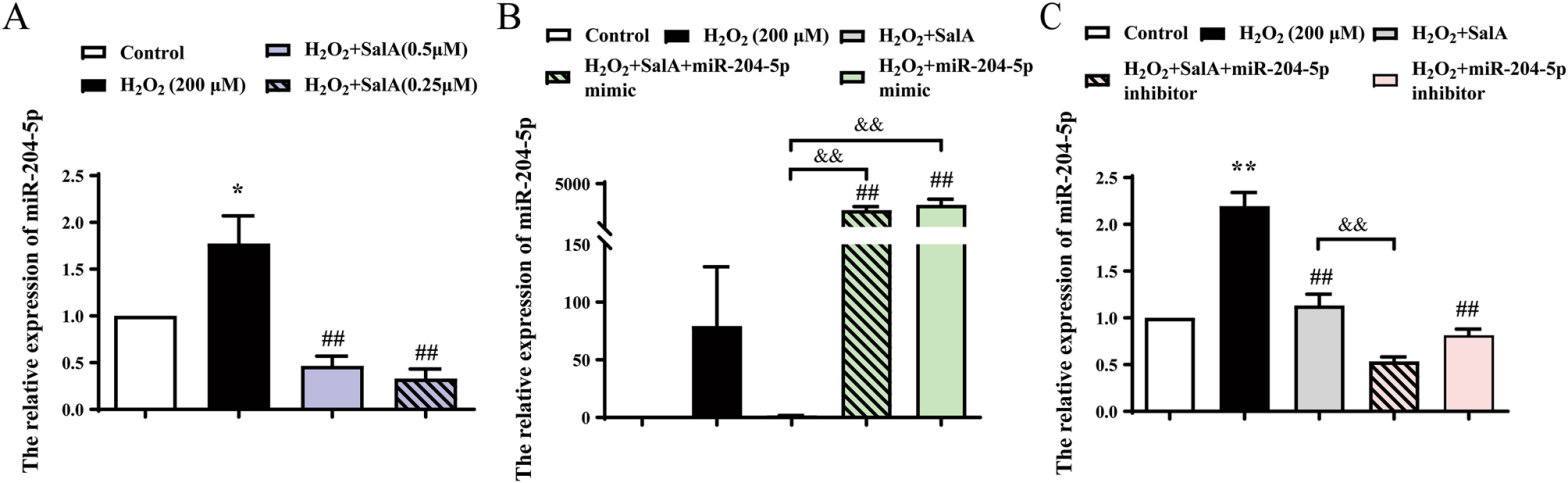
Effects of SalA on miR-204-5p expression of HUVECs. HUVECs were first transfected with miR-204-5p mimic/inhibitor for 6 h, and then cells were induced by 200 μM H_2_O_2_ for 12 h before 24 h SalA treatment. (**A**) The effect of SalA on the miR-204-5p expression of HUVECs induced by H_2_O_2_. (**B**,**C**) The effect of SalA on the miR-204-5p expression of H_2_O_2_-induced HUVECs with miR-204-5p mimic/inhibitor. Values are expressed as the mean ± SD (n = 3) (*P < 0.05, **P < 0.01 vs. Control, ^*##*^*P* < 0.01 vs. H_2_O_2_, ^&&^*P* < 0.01 vs. H_2_O_2_ + SalA).

To determine whether the effect of SalA on the expression of these miR-204-5p was in accordance with our hypothesis, miRNA expression was measured by qRT–PCR (Fig. 5A). Compared with control group, miR-204-5p was up-regulated by H_2_O_2_ inducement. SalA treatment significantly reduced the expression of miR-204-5p (*P* < 0.01) in H_2_O_2_ induced HUVECs.

To further confirm the interaction of SalA and miR-204-5p, miR-204-5p mimic or miR-204-5p inhibitor were transferred into H_2_O_2_-induced HUVECs. Consistent with expected, the high expression of miR-204-5p induced by miR-204-5p mimic transfection reversed the decrease effect of SalA treatment (Fig. 5B); The transfection of miR-204-5p inhibitor downregulated the expression of miR-204-5p (*P* < 0.01) compared with the H_2_O_2_ group, which was similar as the effect of SalA, and miR-204-5p inhibitor enhanced the inhibition effect of SalA on miR-204-5p (*P* < 0.01) (Fig. 5C).

### SalA alleviated H_2_O_2_-induced HUVECs apoptosis though miR-204-5p/p53 pathway

**Figure 6:**
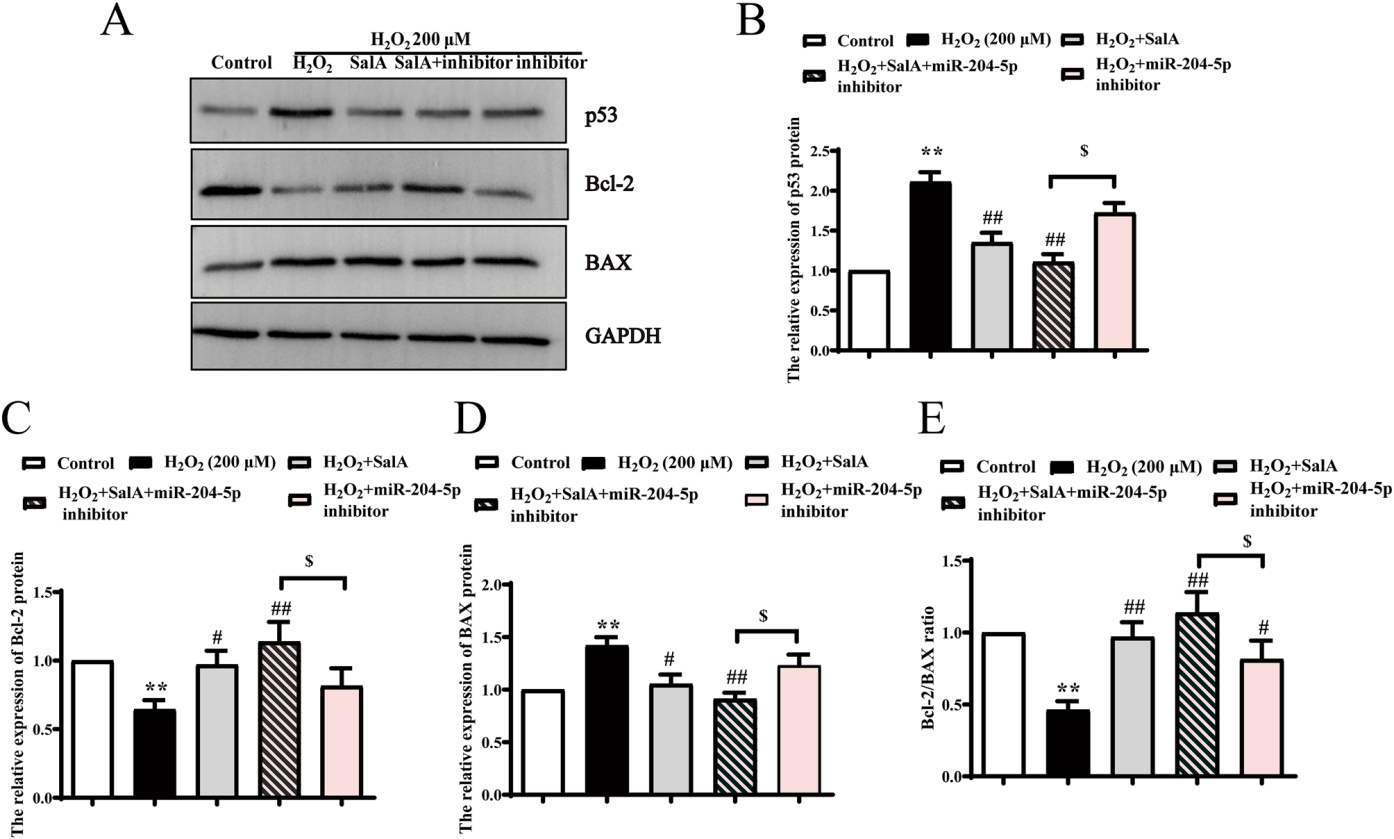
Effects of SalA on expression of apoptosis-related protein in H_2_O_2_-induced HUVECs by inhibiting miR-204-5p. HUVECs were first transfected with miR-204-5p inhibitor for 6 h, and then cells were induced by 200 μM H_2_O_2_ for 12 h before 24 h SalA treatment. (**A**) The effect of SalA on the protein expressions of (**B**) p53, (**C**) Bcl-2 and (**D**) BAX were detected by western blotting. (**E**) The expression ratio of Bcl-2/BAX. Uncropped gel images are provided in the supplementary file. Values are expressed as the mean ± SD (n = 3) (**P < 0.01 vs. Control, ^*#*^*P* < 0.05, ^*##*^*P* < 0.01 vs. H_2_O_2_, ^$^*P* < 0.05 vs. H_2_O_2_ + miR-204-5p inhibitor).

miR-204-5p was found to promote apoptosis by downregulating Bcl-2 in prostate cancer cells^23^. It is thought that p53 causes apoptosis, which is governed by the presence of several apoptotic genes, including Bcl-2 and BAX^24^. Therefore, we were interested in determining the role of miR-204-5p/p53 pathway. The transfection of miR-204-5p inhibitor and SalA has a similar effect on p53, and the combined effect was stronger than on their alone (Fig. 6). Meanwhile, miR-204-5p inhibitor enhanced the effect of SalA on p53 (*P* < 0.05), Bcl-2 (*P* < 0.05), BAX (*P* < 0.05) and Bcl-2/BAX ratio (*P* < 0.05).

## Discussion

Endothelial cells are directly exposed to endogenous danger signals and metabolites in the circulatory system. Endothelial senescence and death associated EC dysfunction are widely recognized to be associated with vascular diseases such as atherosclerosis, hypertension and hyperlipidaemia and is a common early event in CVD^25,26^. Thus, research on EC senescence and death could accelerate the discovery of more effective drugs for the treatment of vascular diseases. In this study, our results demonstrated that SalA reversed the effects of H_2_O_2_-induced on endothelial oxidative injury, EC senescence, apoptosis, autophagy and NLRP3 expression.

The oxidative pathway is one of the important mechanisms associated with cell senescence, and the role of oxidative stress in the initiation and progression of vascular endothelial cell senescence has been widely reported. The excessive production of ROS is one of the prime responses of oxidative stress, result in cell dysfunction, senescence, and even cell death. SOD is an important component of cellular defense against free radical-mediated cellular oxidative damage^27^. In our study, SalA significantly improved cell viability and increased SOD activity after H_2_O_2_-induction, as well as reduced intracellular ROS levels. Consistent with the previous studies^28,29^, SalA reversed the oxidative injury on HUVECs.

Peroxidation products activate transcription factors (including p53) associated with senescence at high levels of oxidative stress^30,31^. It has been found that exposure of cells to oxidative stress leads to senescence by activating p53 through the initiation of the DNA damage response pathway and activating the downstream factor p21. Activated p21 blocks the G1 cell cycle and inhibits the activity of cell cycle proteins, such as Cyclin E1^32^. In our results SalA inhibited p53, p21 and promoted Cyclin E1 expression levels, these results indicated that SalA rescued H_2_O_2_-induced cellular senescence.

Each mode of cell death is associated with the specific cellular signaling cascades. Autophagy is involved in the antioxidant defense mechanism, but excessive or autophagy can be detrimental to cells. LC3 protein is one of the key components of autophagosomes, which regulates the formation and recycling of autophagic vesicles and plays an important role in the initiation and expansion of autophagy. When autophagy is active, LC3 II/I ratio was up-regulated. Pyroptosis is a proinflammatory cell death characterized by the formation of inflammasomes. NLRP3 is a component of the inflammasome, which is activated by specific signals to trigger a series of inflammatory responses and contribute to pyroptosis, thus participating in inflammatory regulation and cell death mechanisms. Apoptosis is one of the most widely studied forms of programmed cell death. There are three classical apoptosis signaling pathways: the extrinsic (death receptor) pathway, the intrinsic (mitochondrion) pathway and the endoplasmic reticulum stress pathway (including ischemia, oxidative stress, hypoxia, etc.). All of which can modulate the expression of the Bcl-2 family of proteins and alter the permeability of the mitochondrial membrane, thereby inducing apoptosis. Our study found that SalA reversed the H_2_O_2_-induced increase in the LC3 II/I ratio and NLRP3 expression, and inhibited HUVECs apoptosis. These results indicating that SalA could alleviate the oxidative stress induced endothelial cell death.

The molecular mechanisms underlying EC senescence or death are not fully understood. In recent years an increasing number of studies on miRNAs as modulators of endothelial dysfunction have provided new insights into endothelial dysfunction, leading to potential new therapeutic approaches. miR-204-5p was reported to regulate apoptosis, autophagy, and cellular senescence by inhibiting target genes in multiple cell types^33–35^. Apoptosis is one of the most common phenotypes regulated by miR-204-5p and its target genes. Bcl-2 is the most common target of miR-204-5p and a key apoptosis regulator by interacting with proapoptotic members of the Bcl-2 family^36,37^. Our data showed that SalA inhibited the expression of miR-204-5p in H_2_O_2_-induced HUVECs, and prompted the expression of Bcl-2.

Notably, when cells are injured, p53 protein acts as a major transcription factor, which is involved in the activation of various factors of the cell cycle, in addition to controlling multiple pathways such as cell senescence, apoptosis, autophagy, etc., and determining the multiple fates of cells under stress conditions^38,39^. Bcl-2 has been confirmed to prevent p53-mediated apoptosis. In addition, p53 has a dual regulatory effect on autophagy^40,41^. It has also been demonstrated that Bcl-2 complex has a key role in the link between autophagy and apoptosis responses^42^. From this it is clear that p53 are important in cellular senescence and cell fate decision. Our results suggested that SalA may target miR-204-5p to regulate the expression of p53.

**Figure 7:**
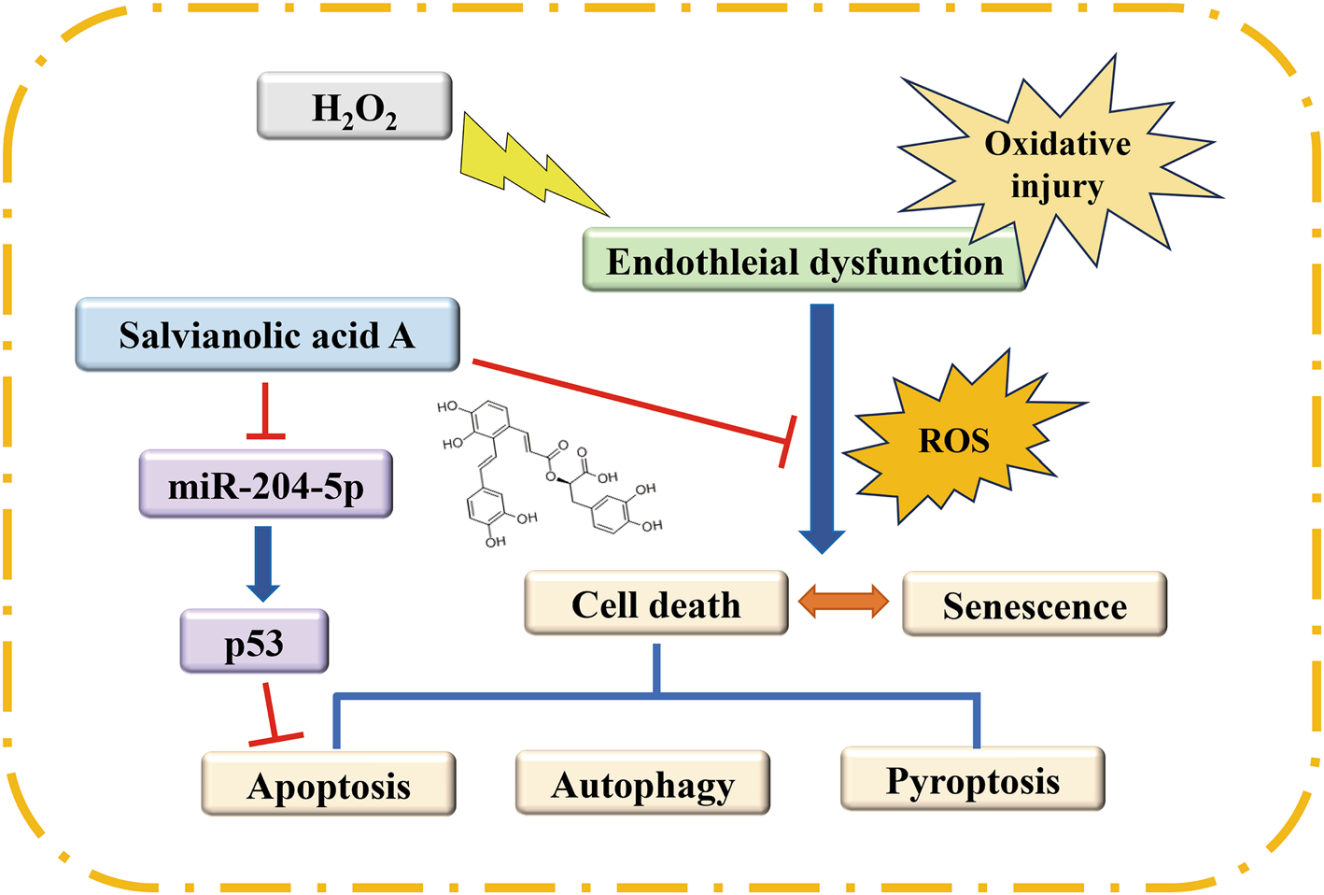
SalA could alleviate H_2_O_2_-induced oxidative injury by downregulating miR-204-5p in HUVECs.

The major limitation of this study is all the experiments were performed based on the cell line and without proper in vivo results. On the other hand, as confirmed by our results, SalA rescues endothelial cell injury by regulating senescence, autophagy, and apoptosis, respectively, but whether there is a correlation between the occurrence of these events still needs to be further explored (Fig. 7). Our future attempts will be to correlate all events using p53 and to clarify the experimental evidence of SalA in vivo.

## Materials and methods

### Materials and chemicals

HUVECs were purchased from Cell Bank of Type Culture collection of Chinese Academy of Sciences (Shanghai, China). Salvianolic acid A (C_26_H_22_O_10_, HPLC ≥ 98%) was purchased from Aladdin Bio-Chem Technology Company (Shanghai, China). has-miR-204-5p mimic/inhibitor were purchased from Guangzhou Ribo Biotechnology Company (Guangzhou, China).

### Cell culture and treatment

HUVECs were cultured in RPMI 1640 medium (Hyclone, USA) supplemented with 10% fetal bovine serum (Biological Industries, USA), 1% Penicillin–Streptomycin at 37 °C in a humidified atmosphere of 5% CO_2_. 200 μM H_2_O_2_ was chosen as the modeling concentration to induce oxidative damage in HUVECs. Cells were exposed to 200 μM H_2_O_2_ for 12 h in serum-free medium following 24 h of normal culture. For SalA treatment, cells were treated with different concentrations of SalA for 24 h after H_2_O_2_ inducing. Cells at passages 3 to 8 were used in all experiments.

### Cell viability assay

Cells (1 × 10^4^ cells/mL, 100 μL/well) were seeded in the 96-well plates with five replicate wells. After treatment, 20 μL Cell Counting Kit-8 reagent (CCK-8, Beyotime, China) was added into each well and incubated at 37 °C for 2 h. The absorbance was measured at 450 nm. Cell viability was defined as a percentage of the control group.

### Cell proliferation detection

After treatments, cell proliferation rate was detected by BeyoClick™ EdU Cell Proliferation Kit with Alexa Fluor 488 (Beyotime, China). In short, cells were incubated with EdU solution at 37 °C for 2 h and fixed with 4% paraformaldehyde at room temperature for 15 min. Then, cells were permeabilized with 0.3% Triton X-100 (prepared with PBS) for 15 min and incubated with the Click reaction solution for 30 min in dark. After, cells were incubated with 1 × Hoechst 33,342 solution at room temperature for 10 min in dark. The ECLIPSE Ti-DH fluorescence microscope (Nikon, Japan) was used to count the numbers of proliferative cells (EdU-positive) in three random fields of view per slide.

### Lactate dehydrogenase (LDH) leakage assay

A LDH assay kit (Beyotime, China) was used to determine the level of extracellular LDH for assessment of cell cytotoxicity. After treatment, the culture medium was collected and centrifugated at 1000 rpm/min for 5 min, the supernatant was employed to add 60 µL LDH working solution following the manufacturer's instructions. The relative LDH release rate was calculated using absorbance at 490 nm.

### Intracellular reactive oxygen species (ROS) assay

A ROS assay kit (Beyotime, China) was used to determine the ROS levels of cells. Cells were incubated with DCFH-DA solution (1:1000, diluted in serum-free RPMI 1640 culture medium) at 37 °C for 20 min. Then cells were washed with serum-free RPMI 1640 medium thrice, and the relative fluorescence intensity of cells was detected within 30 min under fluorescence microscope.

### Superoxide dismutase (SOD) assay

Following the manufacturer’s instructions (Beyotime, China), after treatments, cells were lysed with 50 μL SOD sample preparation solution per well at 4 °C for 15 min and centrifuged at 12,000 rpm/min for 5 min. In a 96-well plate, the supernatant was added to the working solution and incubated at 37 °C for 30 min. The absorbance was determined at 450 nm.

### Apoptosis detection with flow cytometry

Flow cytometry was used to detect cell apoptosis. According to the instructions of Annexin V-FITC/PI Apoptosis Kit (Multi Sciences, China), cells were collected and resuspended by adding 500 μL of 1 × Binding Buffer, and then 5 μL of Annexin V-FITC and 10 μL of PI were added in dark conditions. The apoptotic cells were analyzed by flow cytometry (CytoFlex S, Beckman Coulter, USA).

### miRNA transfection

Cells in the exponential phase of growth were plated in six-well plates at 2 × 10^5^ cells/plate and cultured for 24 h. miR-204-5p mimic/inhibitor (100 nM) and negative controls were transfected to cells for 6 h using Lipofectamine RNAimax transfection reagent (Invitrogen, USA) according to the manufacturer’s protocols. Cells were induced with H_2_O_2_, 24 h after the end of miR-204-5p mimic/inhibitor transfection.

### Western blotting

After treatments, cells were washed with PBS twice and lysed with RIPA lysis buffer (Beyotime, China) on ice, and the protein concentration was determined by BCA Protein assay kit (Beyotime, China). We separated the protein (40 μg/lane) from each sample by SDS-PAGE gel electrophoresis and transferred it to a PVDF membrane (MilliporeSigma, Germany). The membranes were blocked with 5% non‑fat milk blocking buffer for 2 h at room temperature, washed thrice with TBST. Primary antibodies were incubated overnight on PVDF membranes at 4 °C for 12 h: anti-BAX (1:1000; Proteintech, USA); anti-Bcl-2 (1:1000; Proteintech, USA); anti-Cyclin E1 (1:1000; ImmunoWay, USA); anti-p53(1:2000; Proteintech, USA); anti-p21 (1:500; Beyotime, China); anti-LC3 (1:2000; Cell Signaling Technology, USA); anti-NLRP3 (1:1,000; Cell Signaling Technology, USA); and anti-GAPDH (1:2000; ImmunoWay, USA). And then incubated with HRP‑conjugated goat anti‑rabbit (abs20040ss, 1:30,000; Absin, China) or goat anti-mouse antibody (DW0990, 1:50,000; Dawen Biotec., China) at room temperature for 2 h. The ChemiScope 6200 and ChemiScope 3300 detection systems were used to scan immunoblot bands and capture images. Each experiment was repeated three times using the same procedure to obtain an average value. ImageJ software (version 1.53, https://imagej.net) was used to analyze band intensity. GAPDH was used as the loading control.

### Reverse transcription and real-time qPCR

RNA was extracted by Trizol method. After treatment, the cells were washed twice with pre-cooled PBS, and split on ice for 30 min with 1 mL of RNAiso Plus reagent (TAKARA, Japan) added to each well. After adding 200 μL of chloroform, the mixture was centrifuged at 12,000 rpm/min for 15 min at 4 °C. The 400 μL of supernatant was aspirated, and an equal volume of isopropanol was added, and placed at room temperature for 20 min before centrifugation at 12,000 rpm/min and 4 °C for 15 min. Then 1 mL of 75% ethanol was added to each tube, followed by centrifugation at 12,000 rpm/min for 5 min at 4 °C. After discarding the supernatant and drying at room temperature, 50 μL DEPC water was added to dissolve the RNA.

A Mir-XTM miRNA First-Strand Synthesis Kit (TAKARA, Japan) was used for reverse transcription. Real-time PCR was performed using SYBR® Prime Ex Taq TM II (Tli RNase H Plus) (TAKARA, Japan). Each qPCR reaction contained 12.5 μL 2X TB Green Premix Ex Taq II, 0.5 μL primers (10 µM), 0.5 μL 50X ROX Reference Dye, 2 μL cDNA template, and 9 μL ddH_2_O to obtain a final volume of 25 μL. The Step One Plus™ Real-Time PCR System (Applied Biosystems, USA) was used for cDNA amplification and detection. The thermal cycler conditions were as follows: Hold for 10 s at 95 °C, followed by 40 cycles of a two‑step PCR consisting of a 95 °C step for 10 s and a 60 °C step for 20 s. U6 was utilized as a housekeeping gene for normalizing miRNA expression levels. The data were analyzed according to the 2^–ΔΔCt^ method.

The primers were as follows: miR-204-5p: 5′-GCGAGCACAGAATTAATACGC-3′ (forward) and 5′-TCAGTGCACTACAGAACTTTGT-3′ (reverse); U6: 5′-CTCGCTTCGGCAGCACA-3′ (forward) and 5′-AACGCTTCACGAATTTGCGT-3′ (reverse).

### Statistical analysis

If not stated otherwise, results are based on at least three independent experiments (n ≥ 3), and data are presented as mean ± standard deviation (SD) by using GraphPad Prism 8 software (https://www.graphpad.com). For multiple comparisons, the measurement data were subjected to one-way analysis of variance (ANOVA) followed by Tukey post hoc test. *P* < 0.05 and* P* < 0.01 was considered a significant difference.

### Supplementary Information

Supplementary Information.

## Data Availability

All data generated during this study are included in this published article and its supplementary file.
