# Supplementary material for: Salvianolic acid A alleviates H2O2-induced endothelial oxidative injury via miR-204-5p
Source: Sci Rep. 2024 May 24;14:11931. doi: 10.1038/s41598-024-62556-4 (PMC11126572; doi:10.1038/s41598-024-62556-4)

Salvianolic acid A alleviates H_2O_2-induced endothelial oxidative injury via miR-204-5p

Qiao Xilin[1+], Cao Shuyu[1+], Chen Shuaiyu[1+], Guo yan[2], Chen Nipi[1], Zheng Ying[3*], Jin Bo[1*]
[1]School of Life Science, Zhejiang Chinese Medical University, Hangzhou, Zhejiang, China
[2]Hangzhou TCM Hospital Affiliated to Zhejiang Chinese Medical University, Hangzhou, Zhejiang, China
[3]The 903rd Hospital of the People's Liberation Army, Hangzhou, Zhejiang, China

[+]These authors contributed equally to this work.
*Correspondence: Dr Jin Bo, jinbo@zcmu.edu.cn, or Dr Zheng Ying，zhengying72@163.com

Figure 1A , 1B

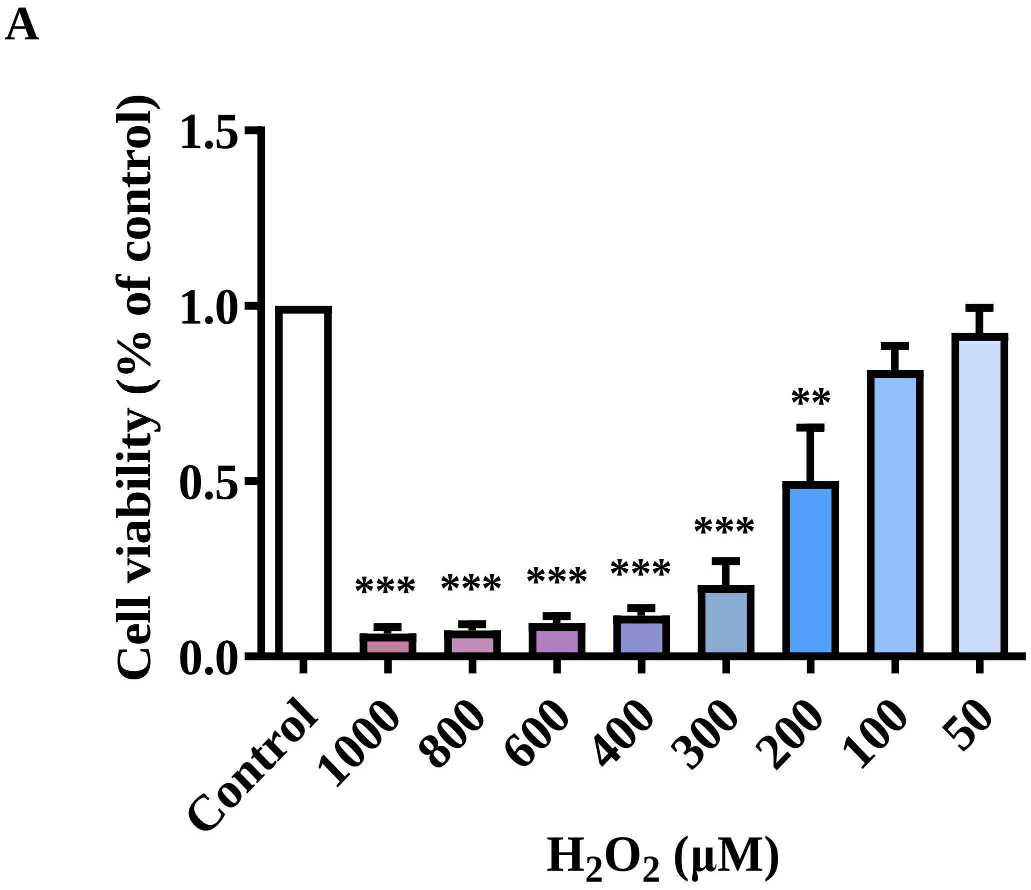

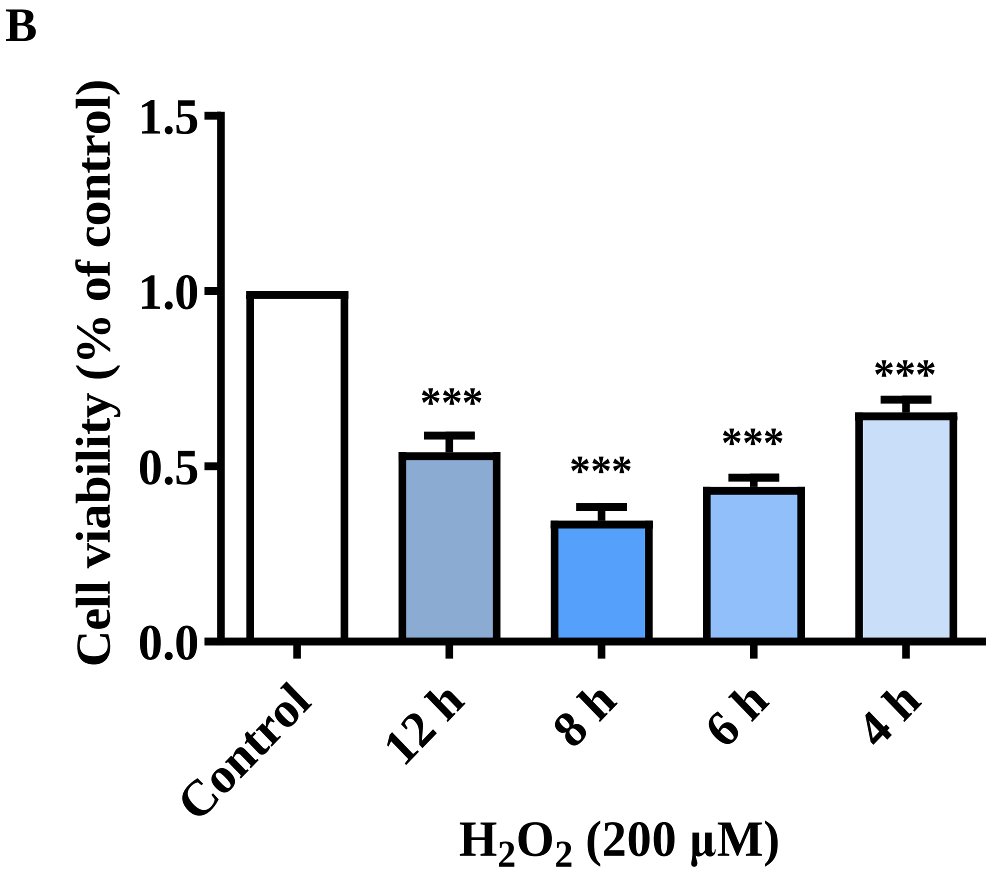

Figure 1A.The effects of different concentration of H_2O_2 (50–1000 μM) treated for 4 h on the cell viability of HUVECs. (*****P<0.01,*** *** ***P< 0.001*** **vs Control)**

Figure 1B. The effects of different treatment time of 200 μM H_2O_2 on the cell viability of HUVECs. (******P< 0.001*** **vs Control)**

Figure 1C, 1D

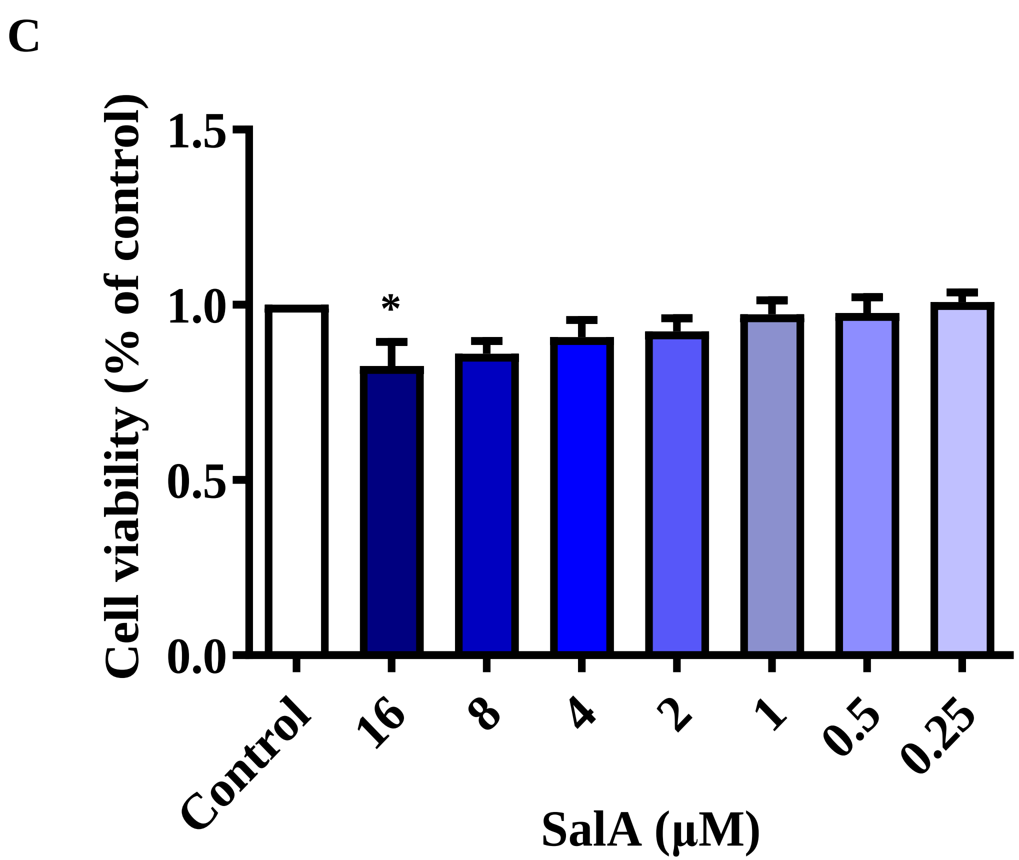

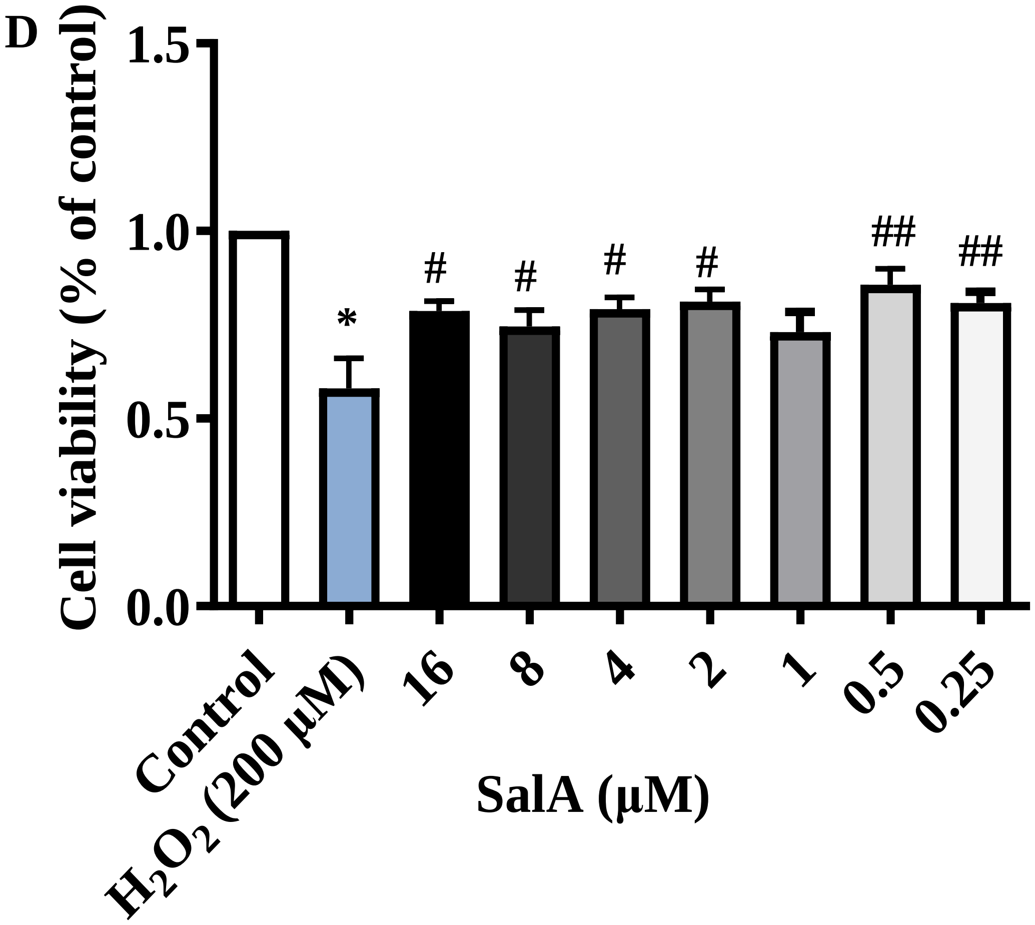

Figure 1C.The effect of different concentration of SalA (0.25–16 μM) on the cell viability of HUVECs. (P< 0.05* vs Control)**

Figure 1D.The effect of different concentration of SalA (0.25–16 μM) on the cell viability in H_2O_2-induced HUVECs. (P<0.05* vs Control, *#P<0.05*, *##P<0.01* vs H_2O_2)**

Figure 1E

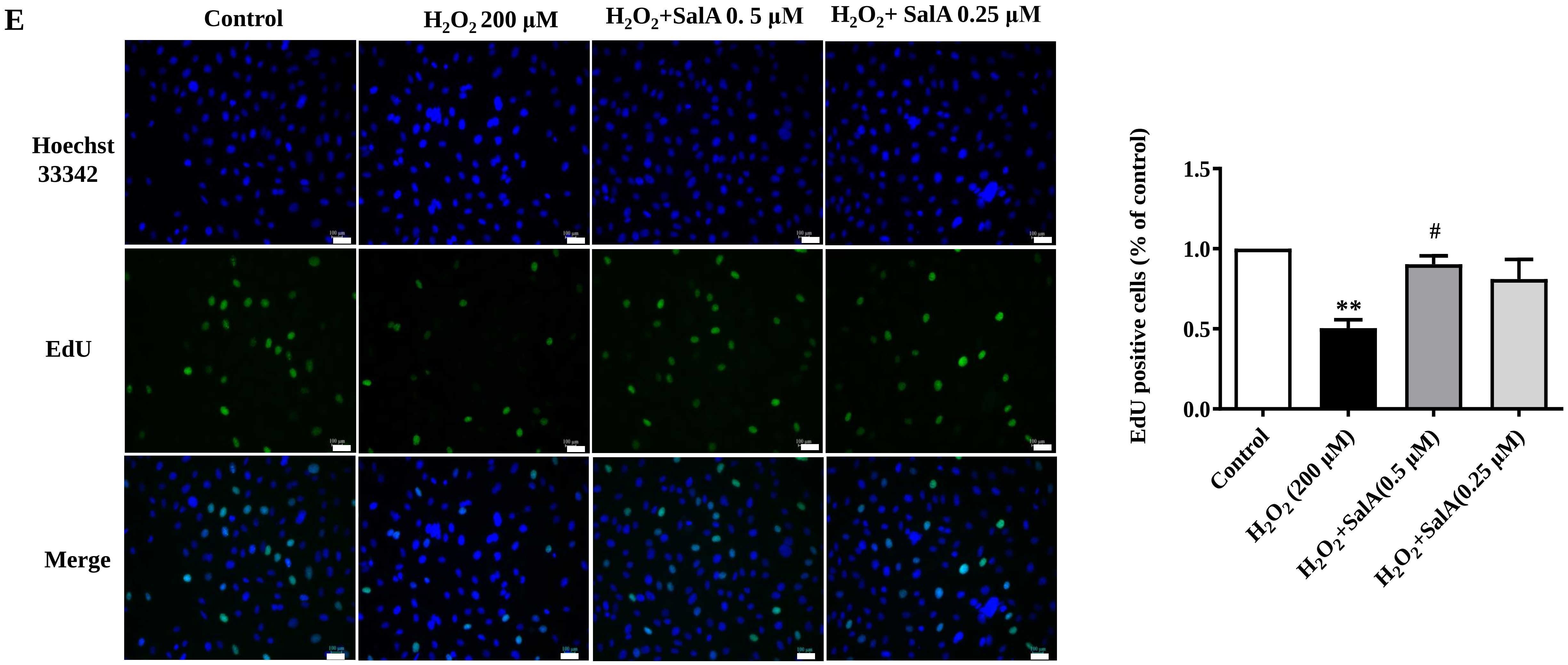

Figure 1E.The effect of SalA on the cell proliferation rate in H_2O_2-induced HUVECs.

(*****P<0.01*** **vs Control,** $^{\#}$***P<0.05*** **vs** H_2O_2**).**

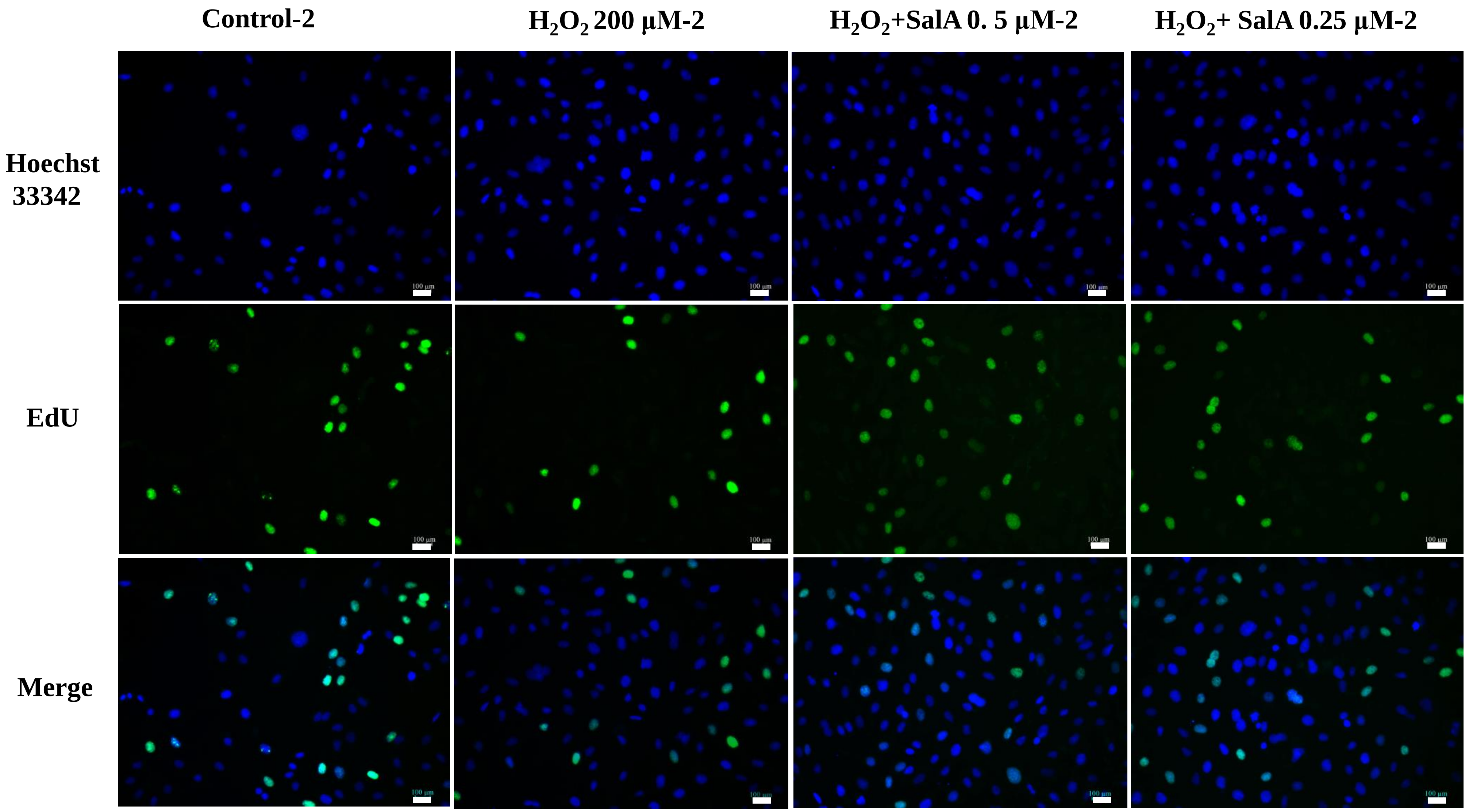

Control-2
H_2O_2 200 μM-2
H_2O_2+SalA 0. 5 μM-2
H_2O_2+ SalA 0.25 μM-2
Hoechst 33342
EdU
Merge

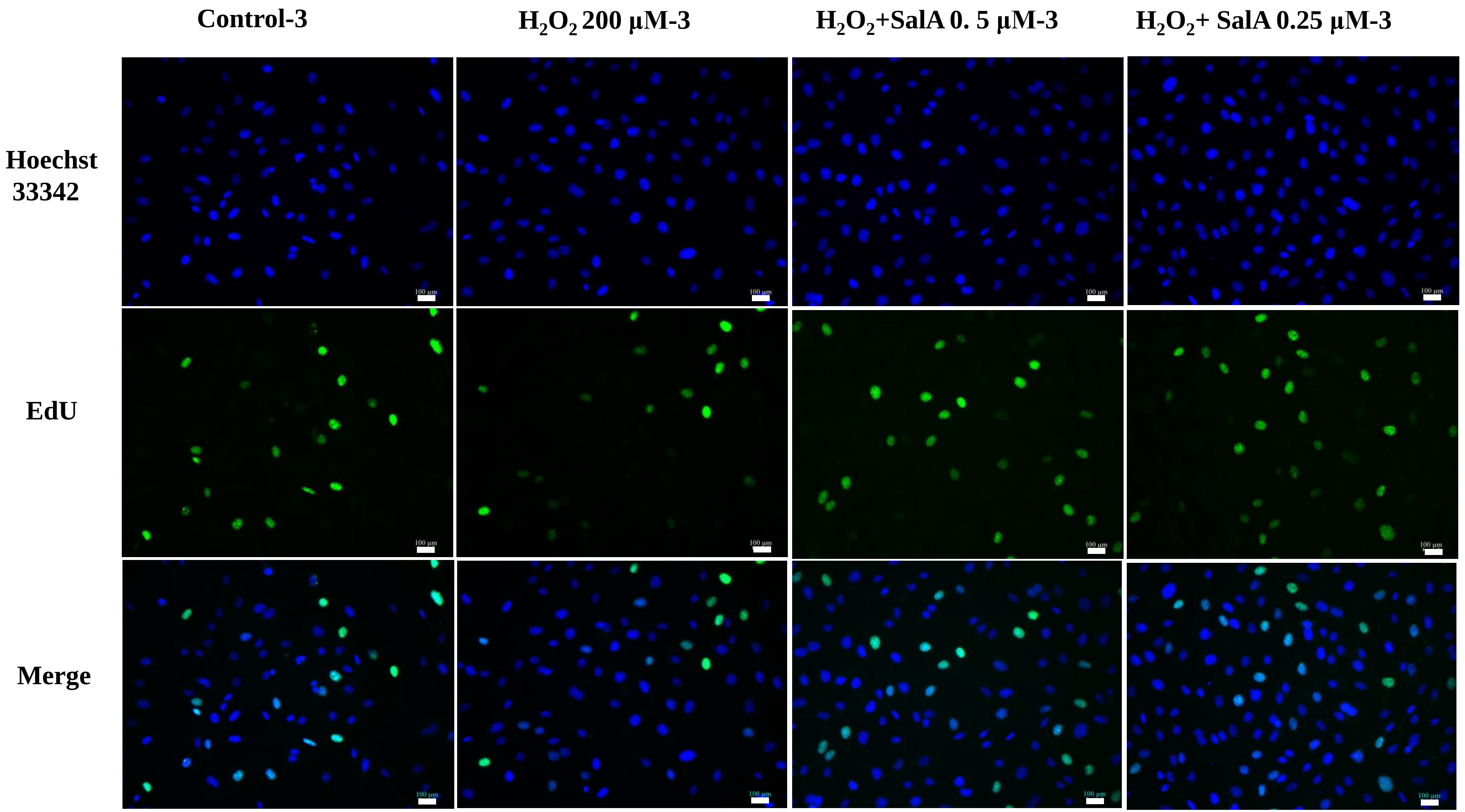

Control-3
H2O2 200 μM-3
H2O2+SalA 0. 5 μM-3
H2O2+ SalA 0.25 μM-3
Hoechst 33342
EdU
Merge
100 μm

Figure 1F

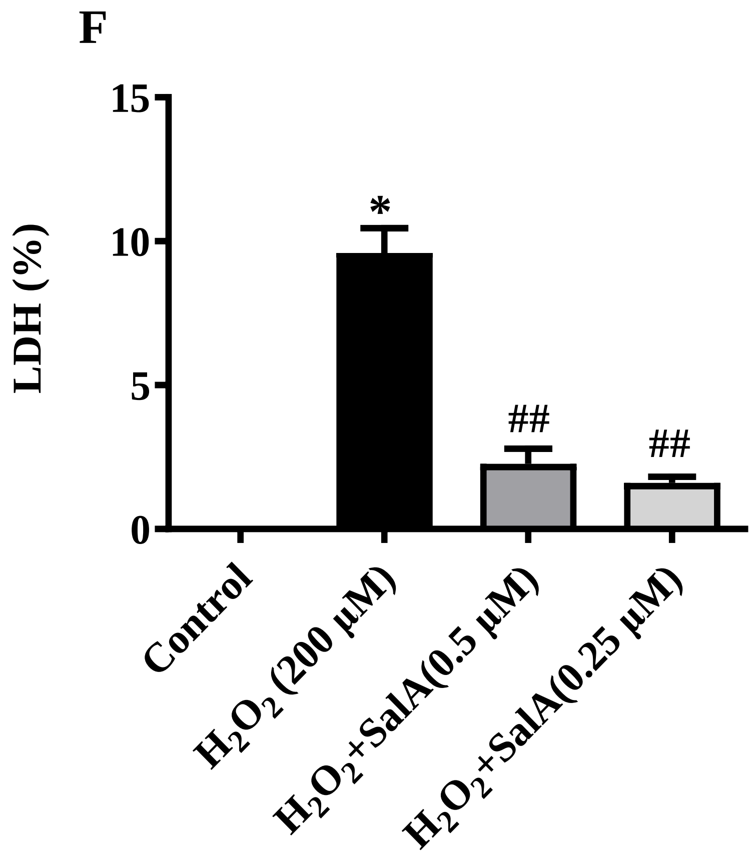

Figure 1F.The effect of SalA on LDH release in H_2O_2-induced HUVECs.

(*$P<0.05$ vs Control,##$P<0.01$ vs H_2O_2).

Figure 2A

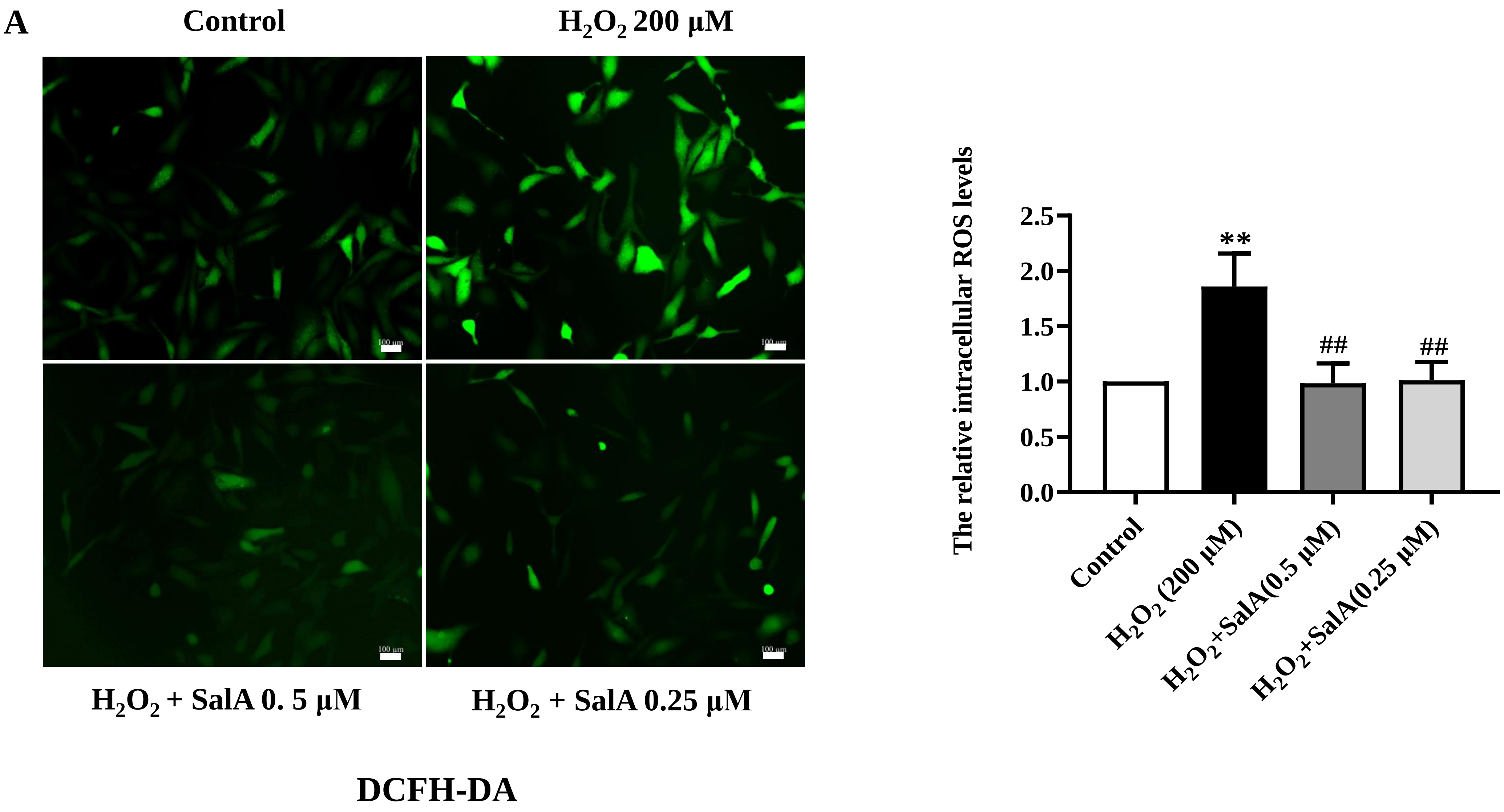

Figure 2A.The effect of SalA on ROS level in H_2O_2-induced HUVECs.

(*P<0.01* vs Control, ##*P<0.01* vs H_2O_2).**

Control-2 **H_2O_2 200 μM-2**

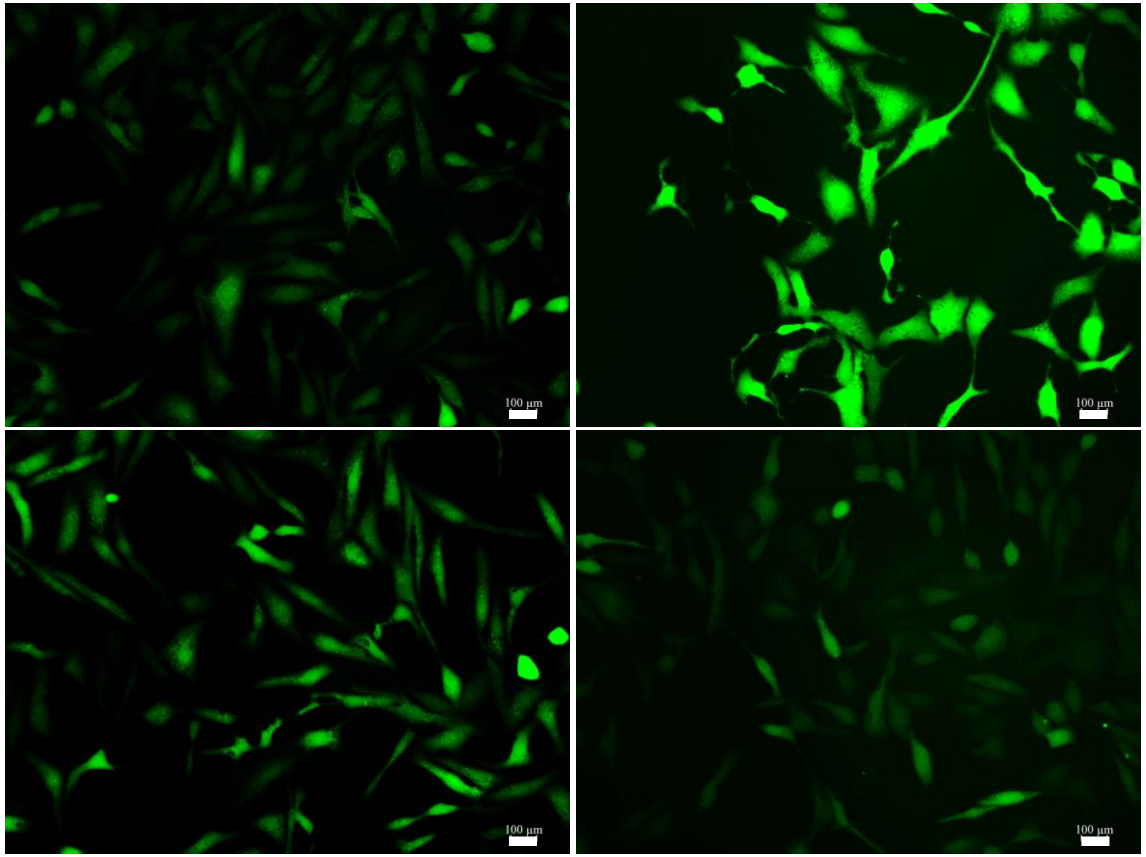

H_2O_2 + SalA 0. 5 μM-2 **H_2O_2 + SalA 0.25 μM-2**

DCFH-DA

Control-3 **H_2O_2 200 μM-3**

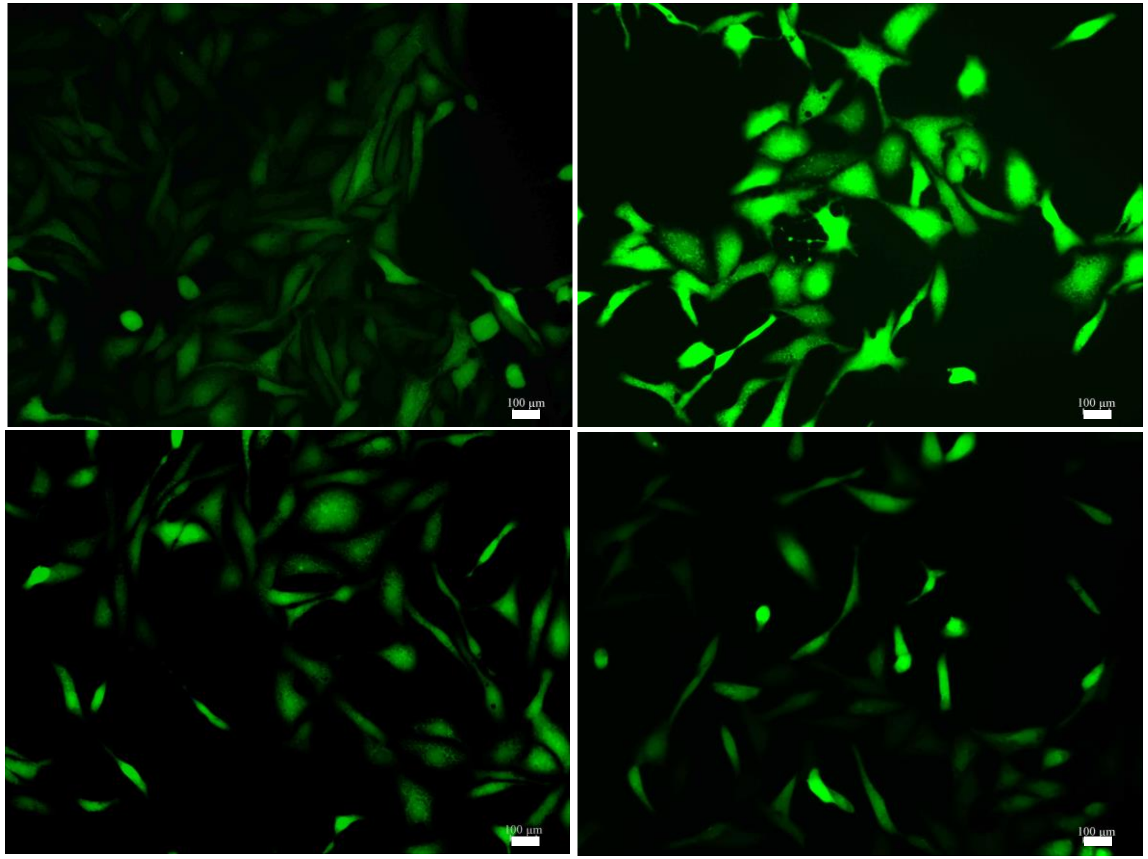

H_2O_2 + SalA 0. 5 μM-3 **H_2O_2 + SalA 0.25 μM-3**

DCFH-DA

Figure 2B

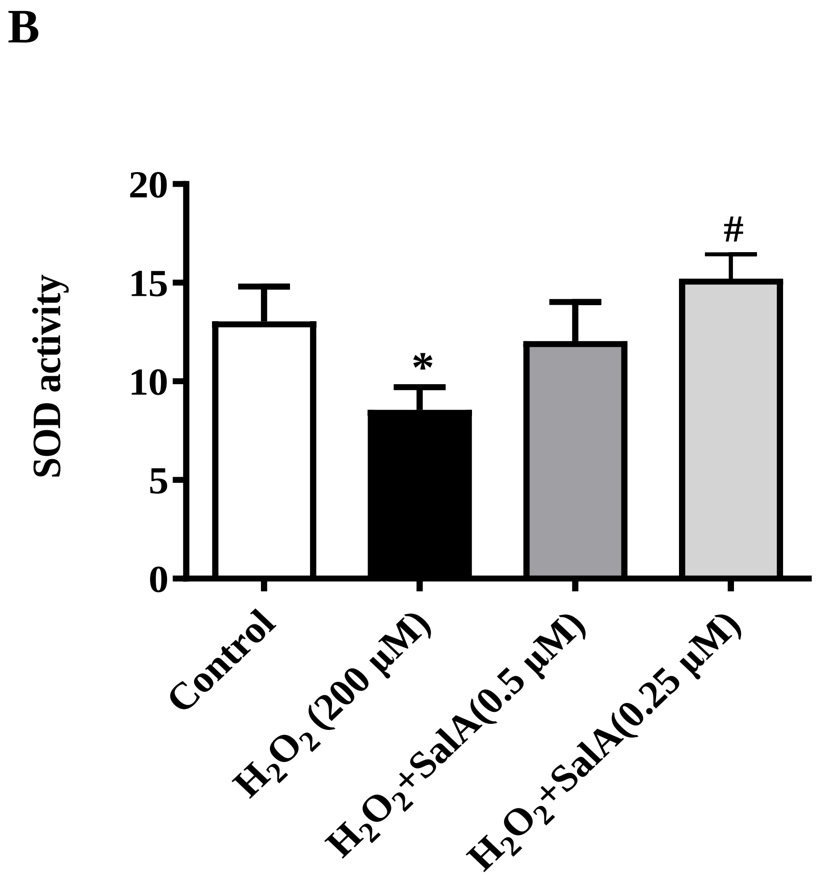

Figure 2B.The effect of SalA on SOD activity in H_2O_2-induced HUVECs.

(*$P<0.05$* vs Control, #*$P<0.05$* vs H_2O_2).

Figure 3

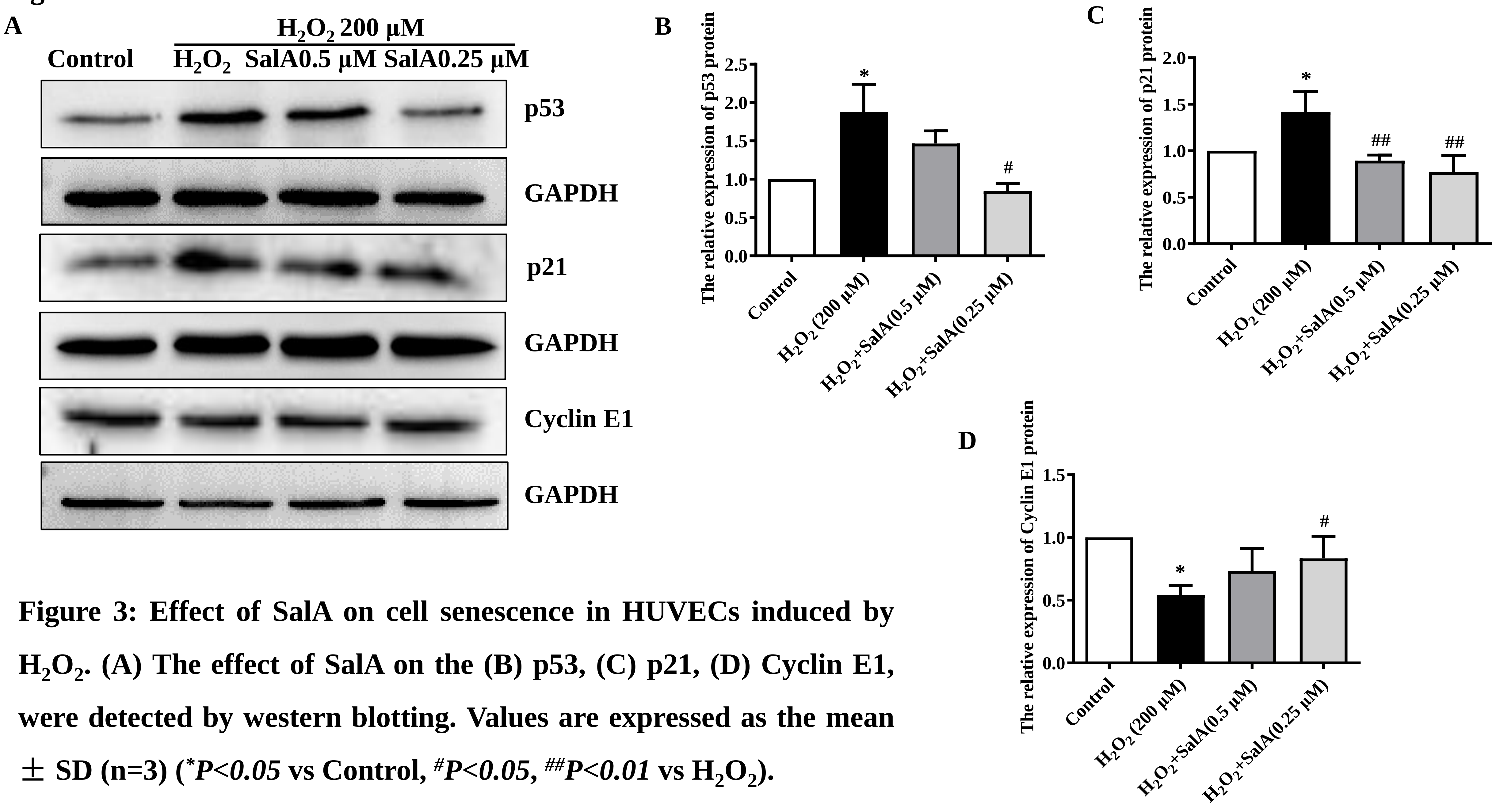

Figure 3: Effect of SalA on cell senescence in HUVECs induced by H_2O_2. (A) The effect of SalA on the (B) p53, (C) p21, (D) Cyclin E1, were detected by western blotting. Values are expressed as the mean ± SD (n=3) (**P<0.05* vs Control, $^{\#}$*P<0.05*, $^{\#\#}$*P<0.01* vs H_2O_2).

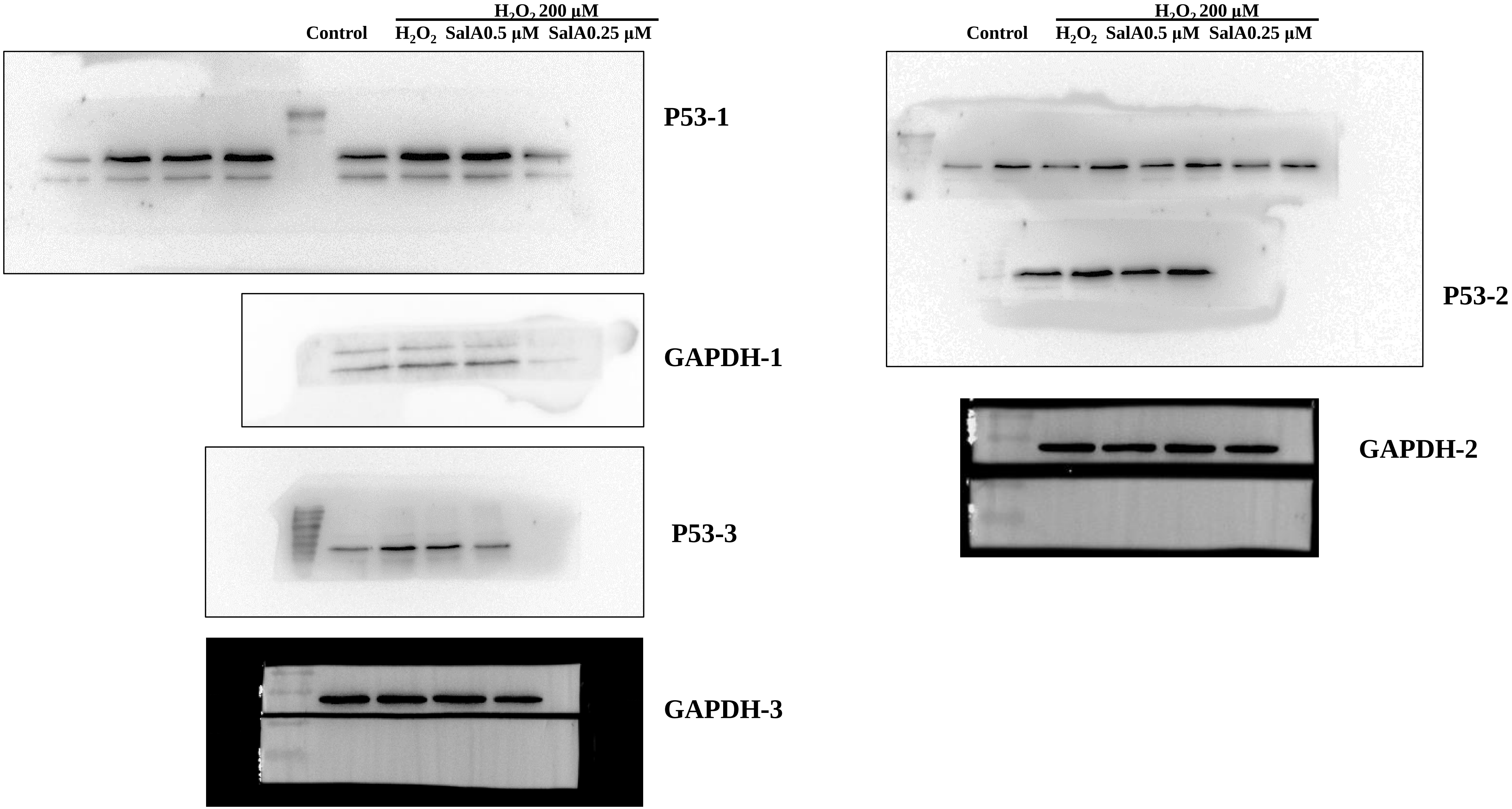

H2O2 200 μM
Control
H2O2
SalA0.5 μM
SalA0.25 μM
P53-1
GAPDH-1
P53-3
GAPDH-3
H2O2 200 μM
Control
H2O2
SalA0.5 μM
SalA0.25 μM
P53-2
GAPDH-2

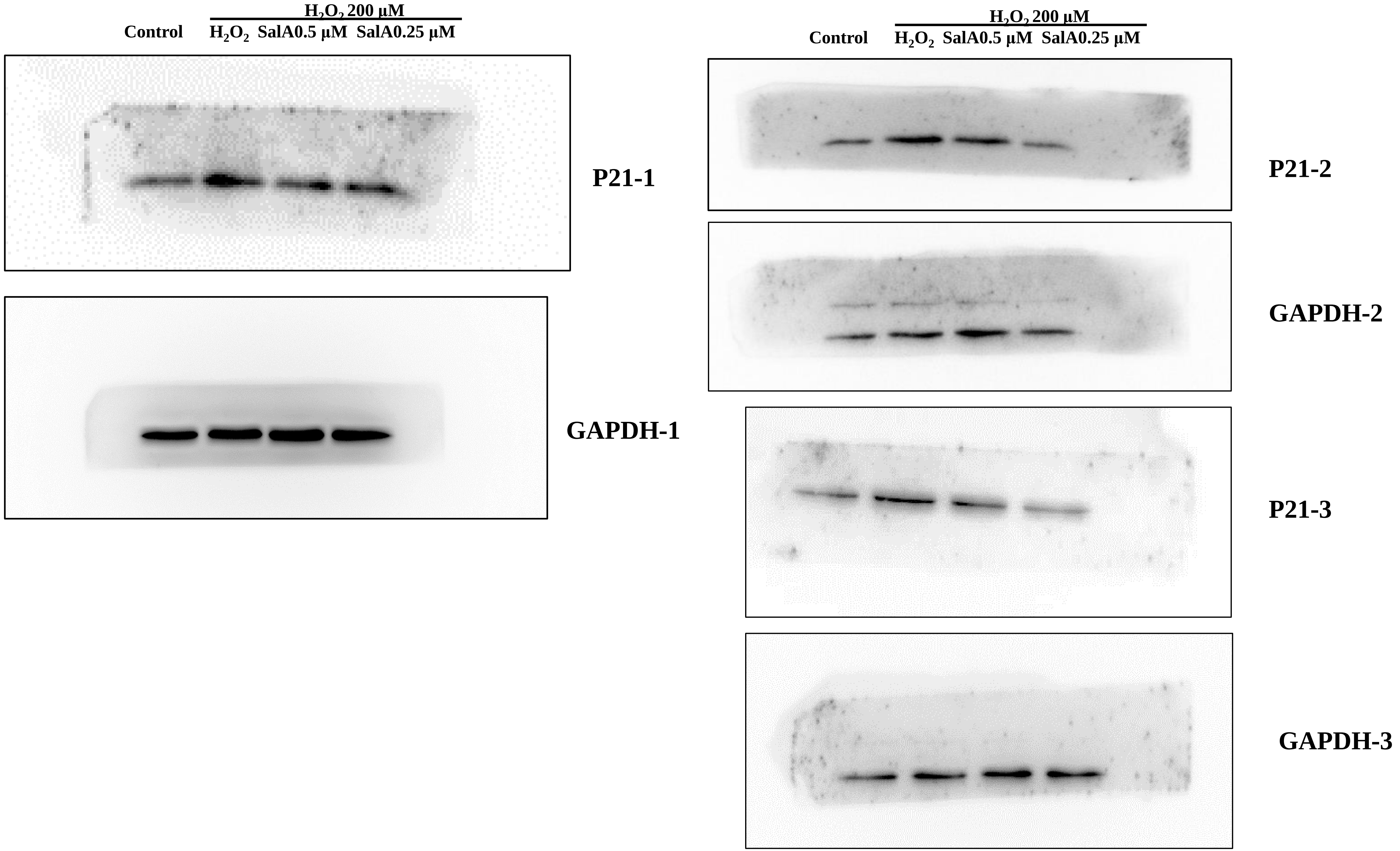
H2O2 200 μM
Control
H2O2
SalA0.5 μM
SalA0.25 μM
P21-1
GAPDH-1
H2O2 200 μM
Control
H2O2
SalA0.5 μM
SalA0.25 μM
P21-2
GAPDH-2
P21-3
GAPDH-3

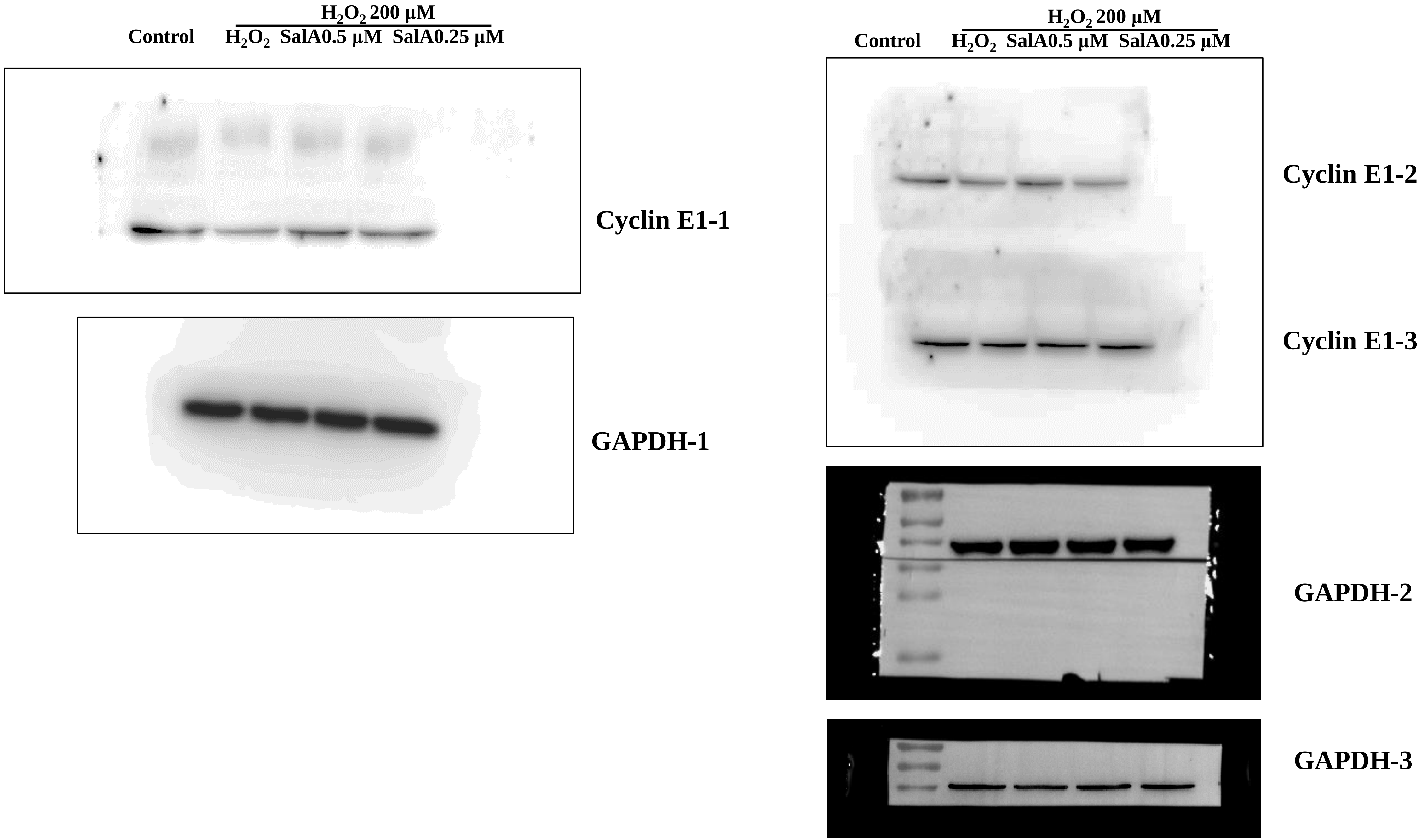

H_2O_2 200 μM
Control
H_2O_2
SalA0.5 μM
SalA0.25 μM
Cyclin E1-1
GAPDH-1
H_2O_2 200 μM
Control
H_2O_2
SalA0.5 μM
SalA0.25 μM
Cyclin E1-2
Cyclin E1-3
GAPDH-2
GAPDH-3

Figure 4A-4D

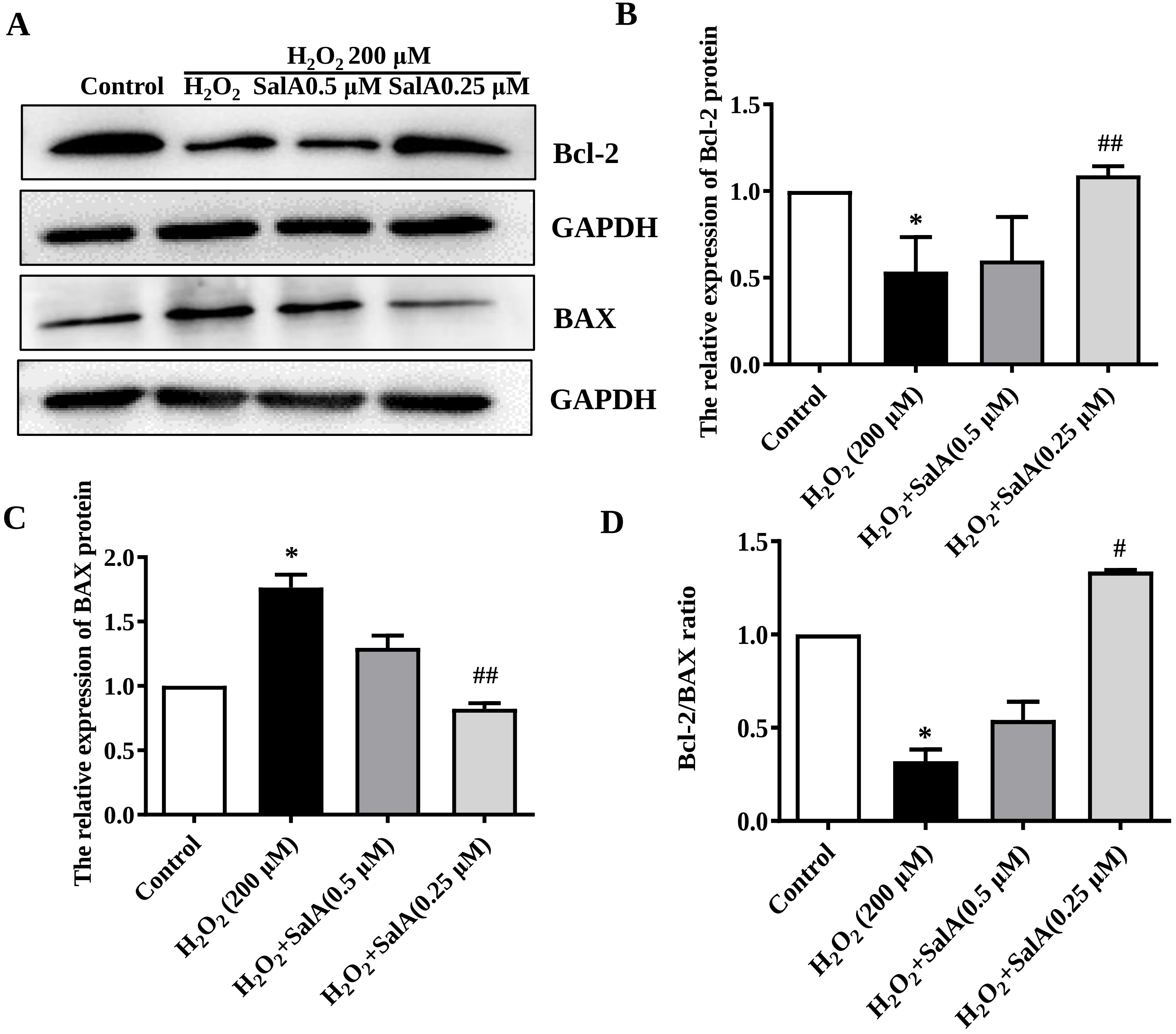

Figure 4A-4D: Effect of SalA on cell apoptosis in HUVECs induced by H_2O_2.

(A) The effect of SalA on the protein expression of (B) Bcl-2 and (C) BAX were detected by western blotting. (D) The expression ratio of Bcl-2/BAX. (**P<0.05* vs Control, $^{\#}$*P< 0.05*, $^{\#\#}$*P<0.01* vs H_2O_2).

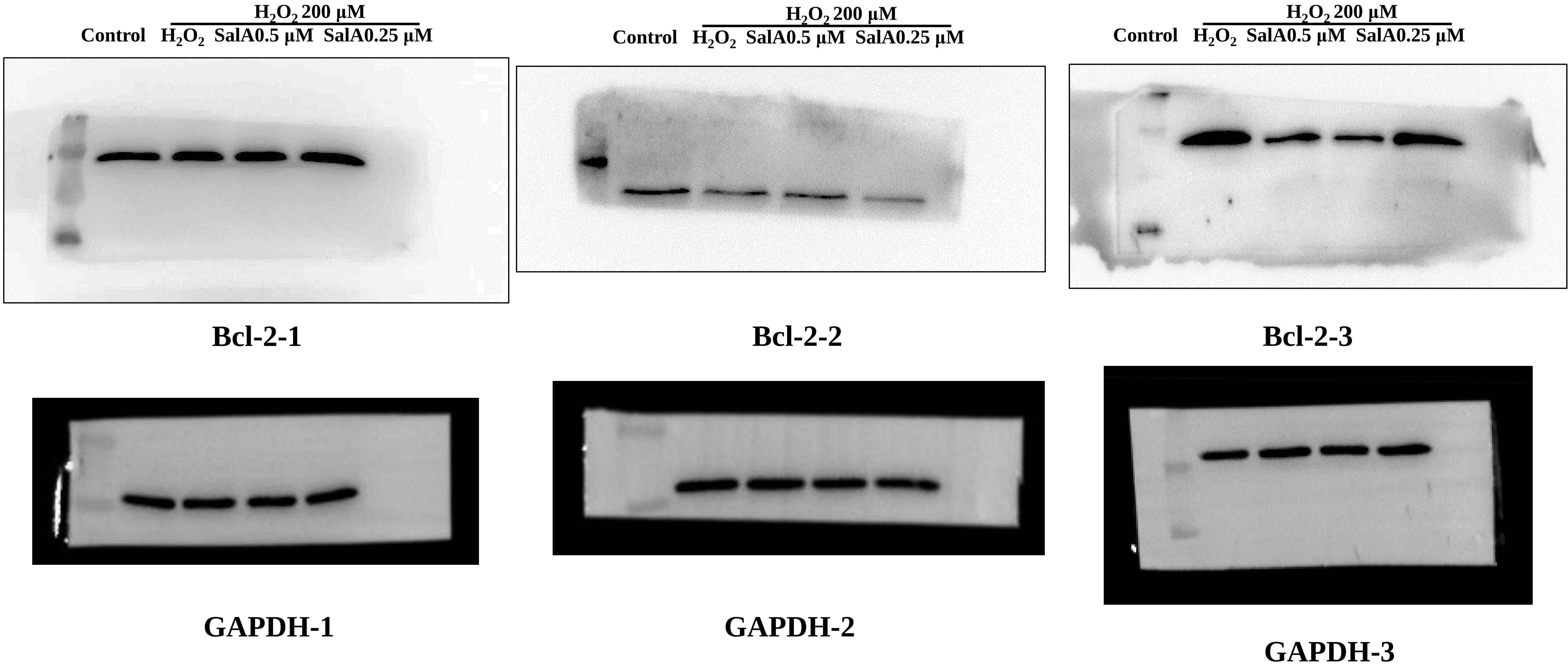
H_2O_2 200 μM
Control
H_2O_2
SalA0.5 μM
SalA0.25 μM
Bcl-2-1
Bcl-2-2
Bcl-2-3
GAPDH-1
GAPDH-2
GAPDH-3

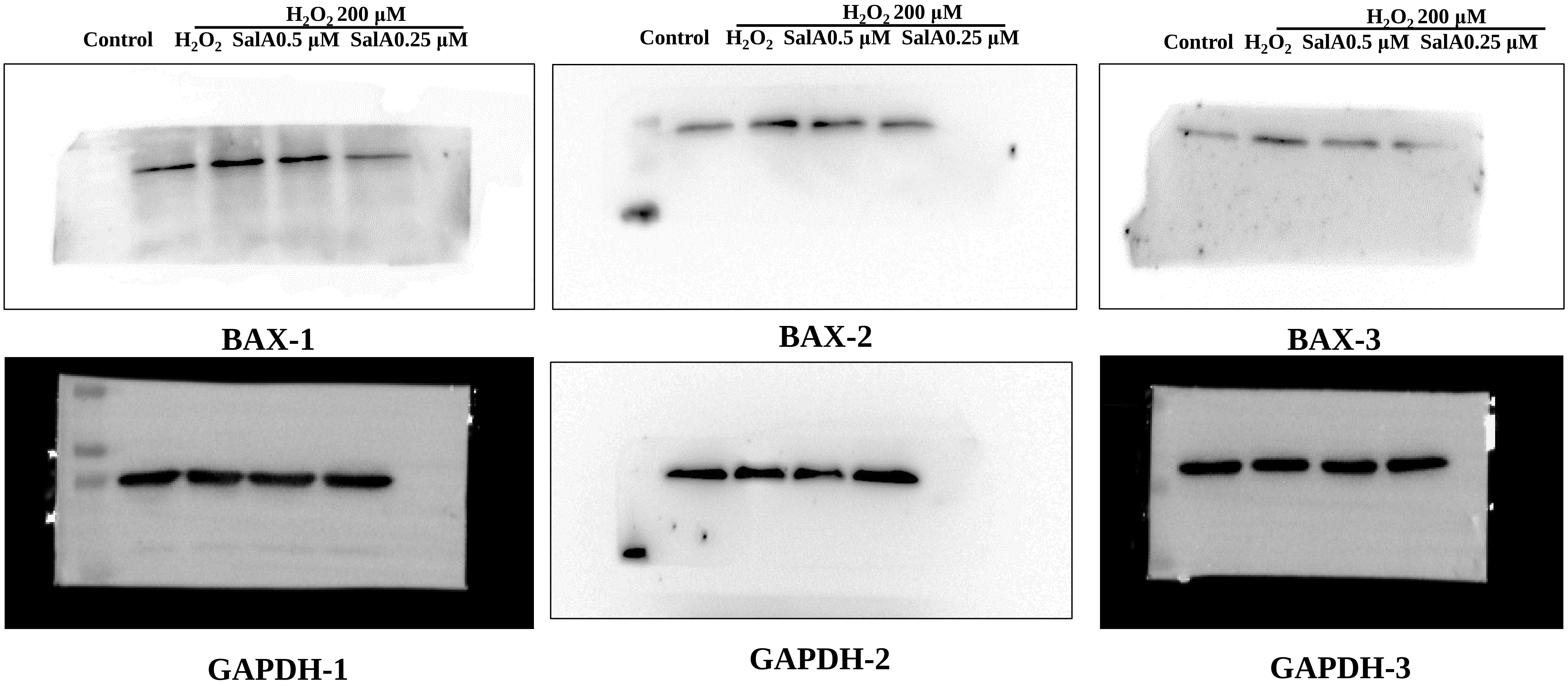
H2O2 200 μM
Control H2O2 SalA0.5 μM SalA0.25 μM
H2O2 200 μM
Control H2O2 SalA0.5 μM SalA0.25 μM
H2O2 200 μM
Control H2O2 SalA0.5 μM SalA0.25 μM
BAX-1
BAX-2
BAX-3
GAPDH-1
GAPDH-2
GAPDH-3

Figure 4E-4F

E

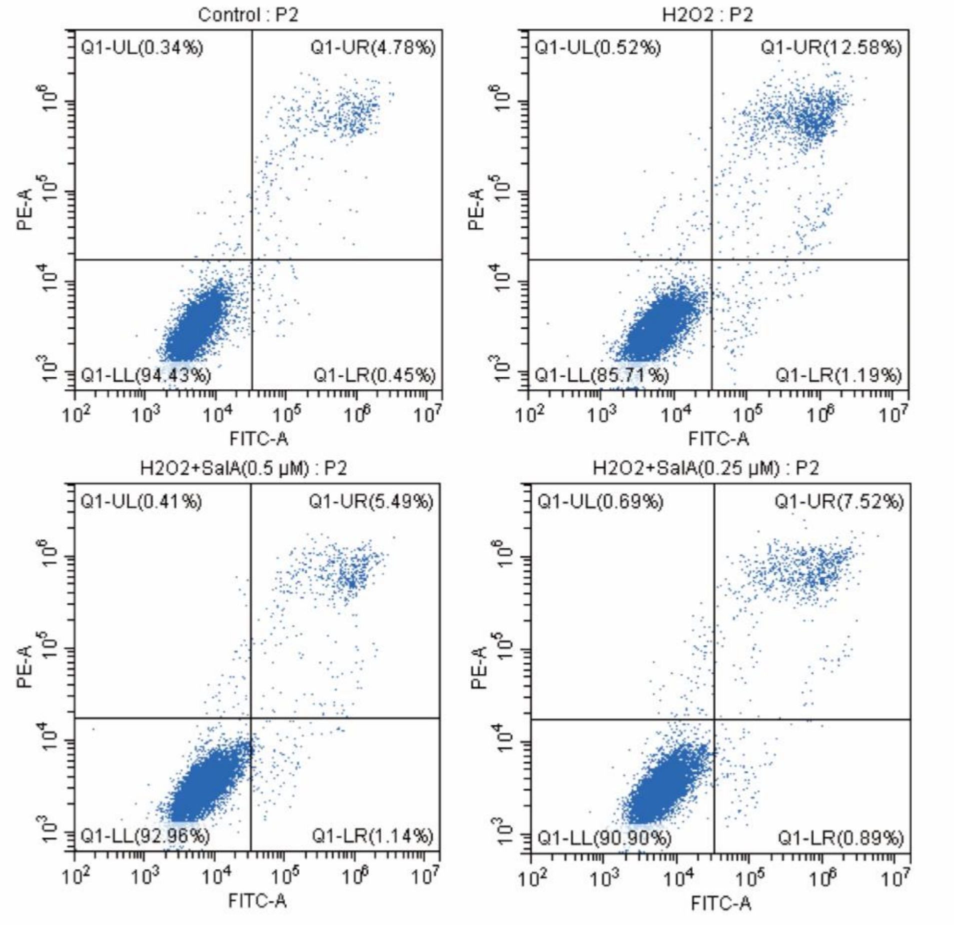

F

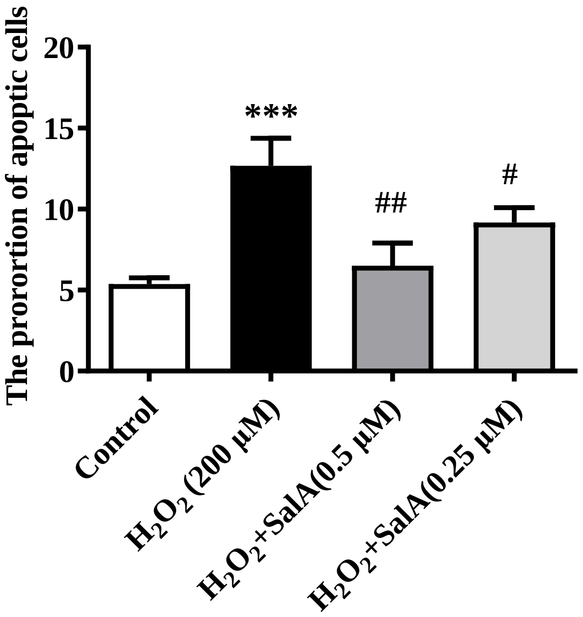

Figure 4E-4F:

(E-F) Representative images of flow cytometry analysis of the effect of SalA on cell apoptosis rate. (****P<0.001* vs Control, #*P<0.05*, ##*P<0.01* vs H_2O_2).**

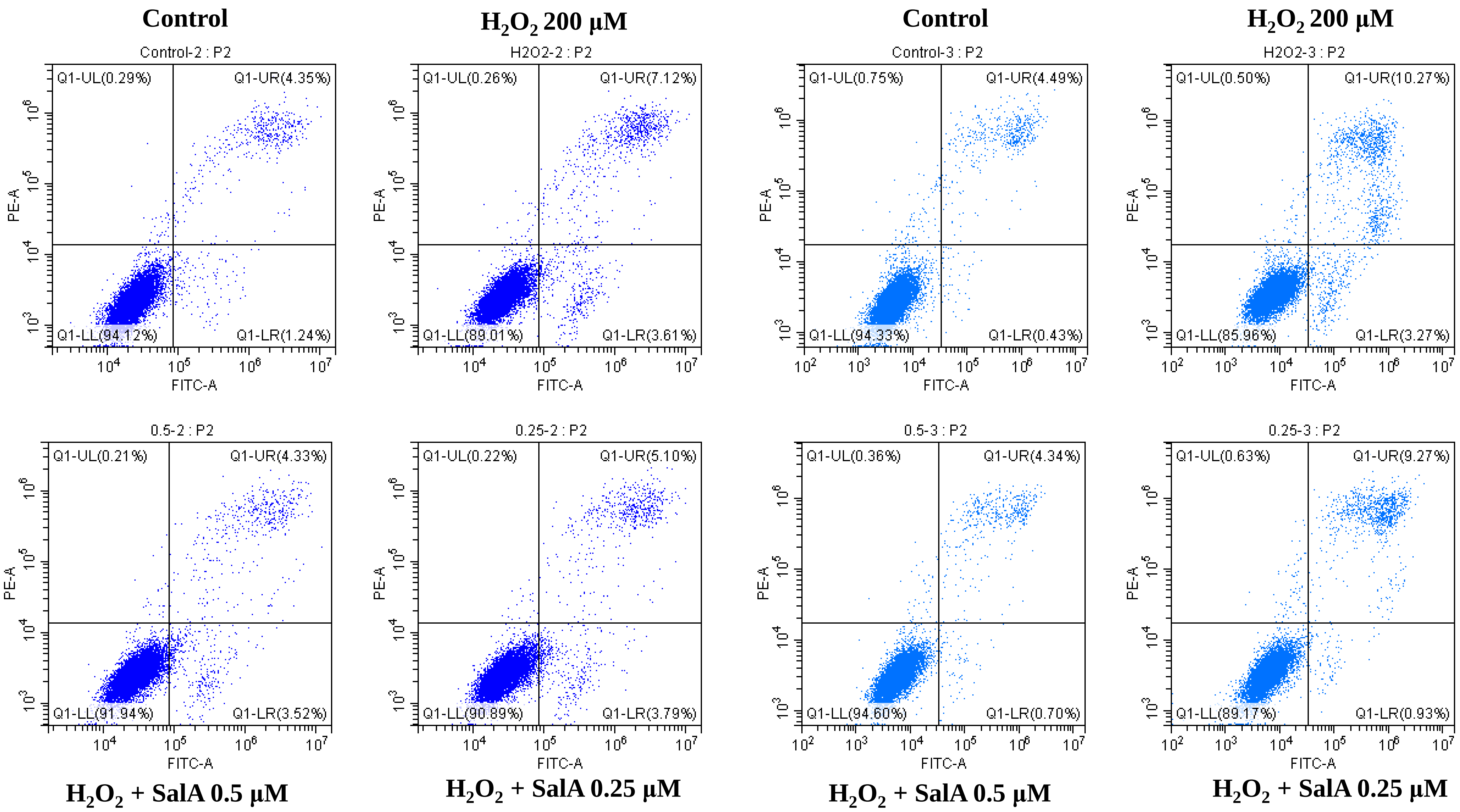
Control
H2O2 200 μM
Control
H2O2 200 μM
Control-2 : P2
Q1-UL(0.29%)
Q1-UR(4.35%)
Q1-LL(94.12%)
Q1-LR(1.24%)
H2O2-2 : P2
Q1-UL(0.26%)
Q1-UR(7.12%)
Q1-LL(89.01%)
Q1-LR(3.61%)
Control-3 : P2
Q1-UL(0.75%)
Q1-UR(4.49%)
Q1-LL(94.33%)
Q1-LR(0.43%)
H2O2-3 : P2
Q1-UL(0.50%)
Q1-UR(10.27%)
Q1-LL(85.96%)
Q1-LR(3.27%)
0.5-2 : P2
Q1-UL(0.21%)
Q1-UR(4.33%)
Q1-LL(91.94%)
Q1-LR(3.52%)
0.25-2 : P2
Q1-UL(0.22%)
Q1-UR(5.10%)
Q1-LL(90.89%)
Q1-LR(3.79%)
0.5-3 : P2
Q1-UL(0.36%)
Q1-UR(4.34%)
Q1-LL(94.60%)
Q1-LR(0.70%)
0.25-3 : P2
Q1-UL(0.63%)
Q1-UR(9.27%)
Q1-LL(89.17%)
Q1-LR(0.93%)
PE-A
FITC-A
H2O2 + SalA 0.5 μM
H2O2 + SalA 0.25 μM
H2O2 + SalA 0.5 μM
H2O2 + SalA 0.25 μM

Figure 4G-4I

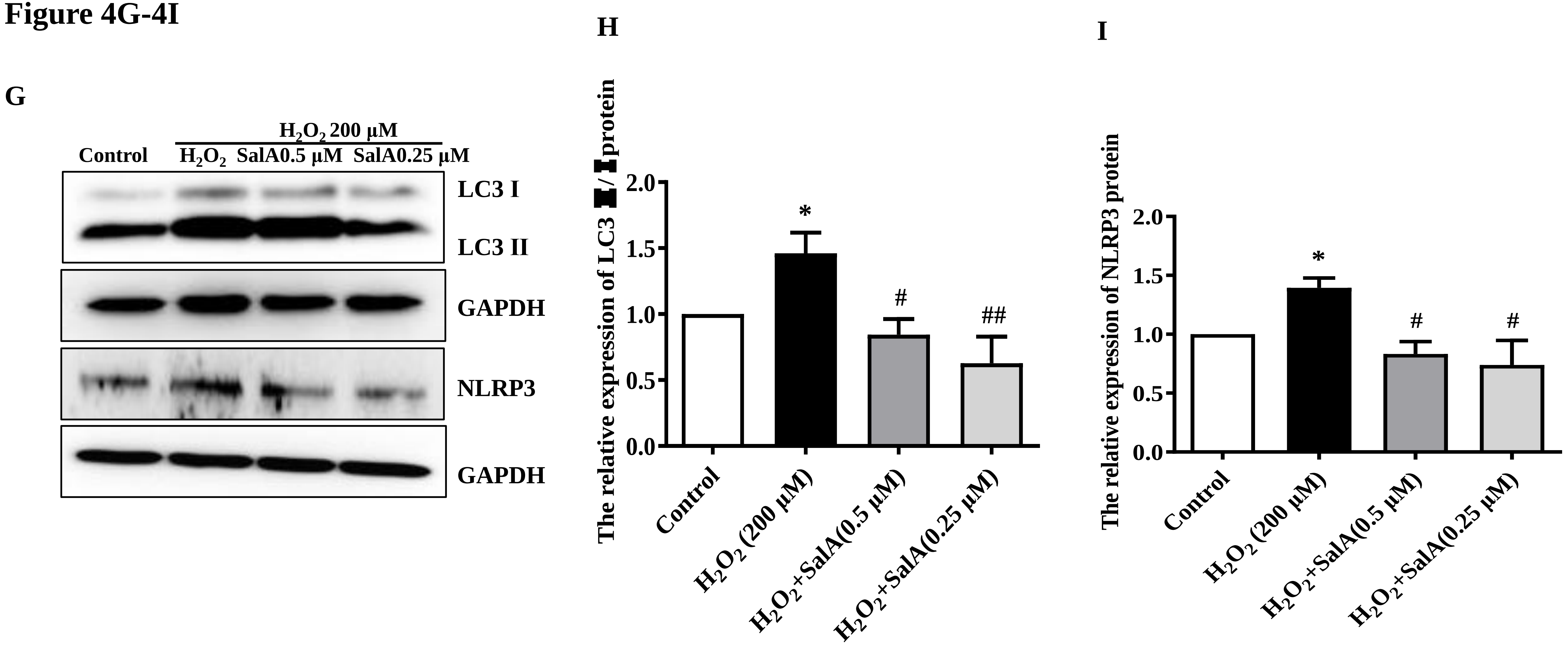

Figure 4G-4I : (G) The effect of SalA on the (H) LC3 II/I ratio and (I) NLRP3 protein expressions were detected by western blotting. (**P<0.05* vs Control, $^{\#}$*P<0.05*, $^{\#\#}$*P<0.01* vs H_2O_2).

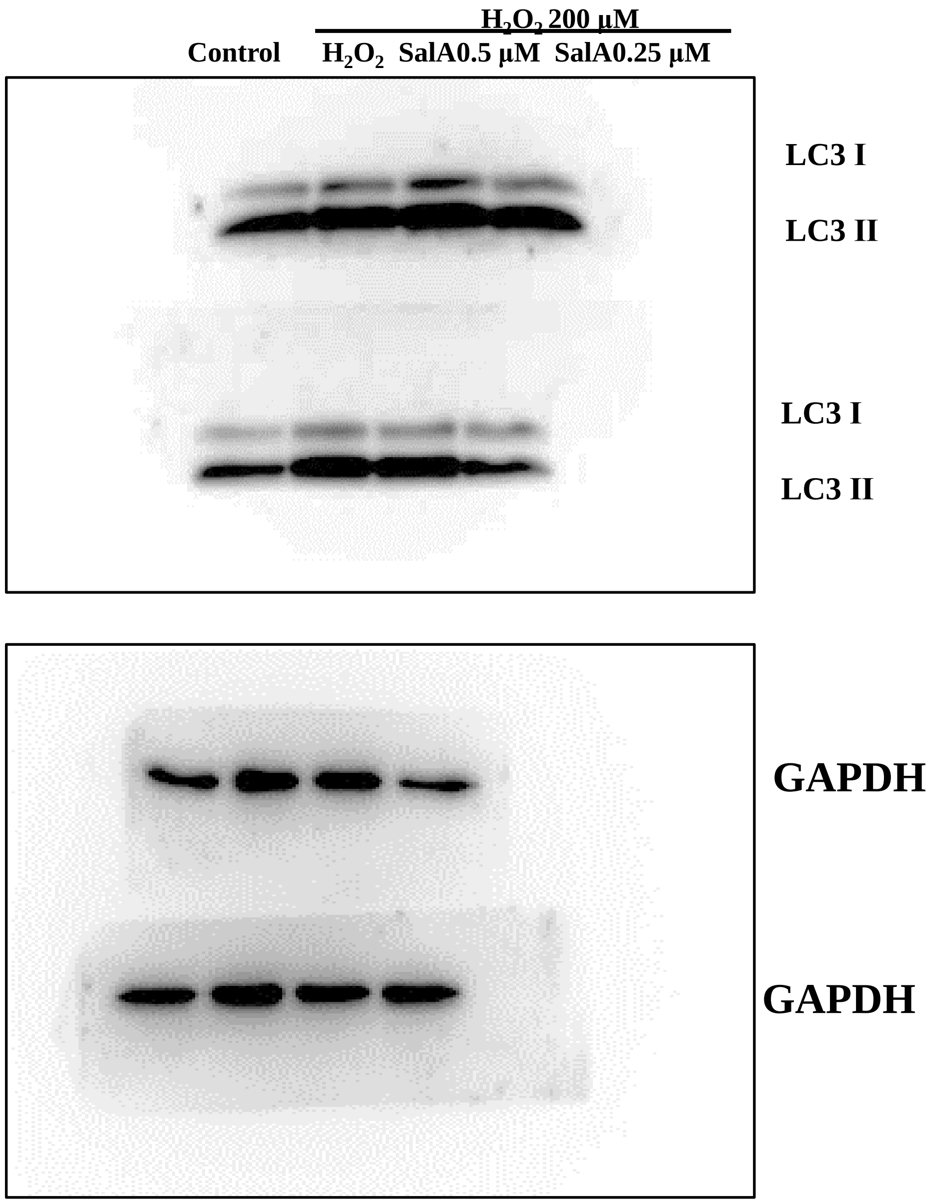
H2O2 200 µM
Control
H2O2
SalA0.5 µM
SalA0.25 µM
LC3 I
LC3 II
LC3 I
LC3 II
GAPDH
GAPDH

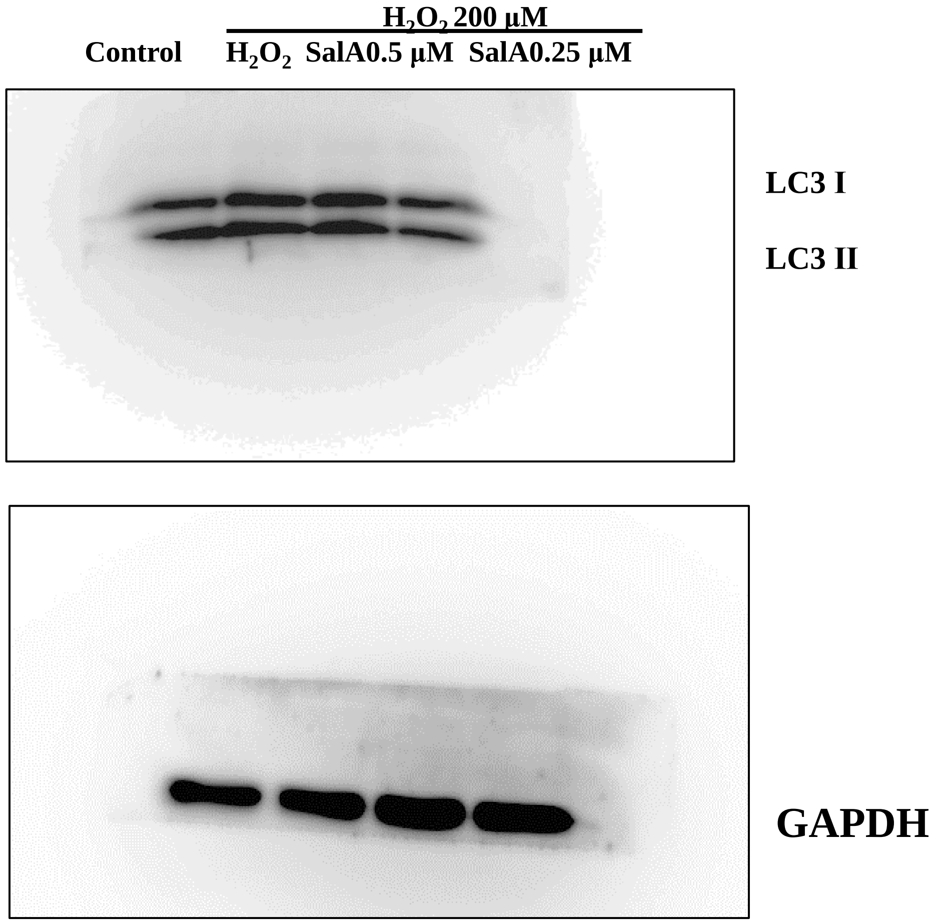
H2O2 200 µM
Control
H2O2
SalA0.5 µM
SalA0.25 µM
LC3 I
LC3 II
GAPDH

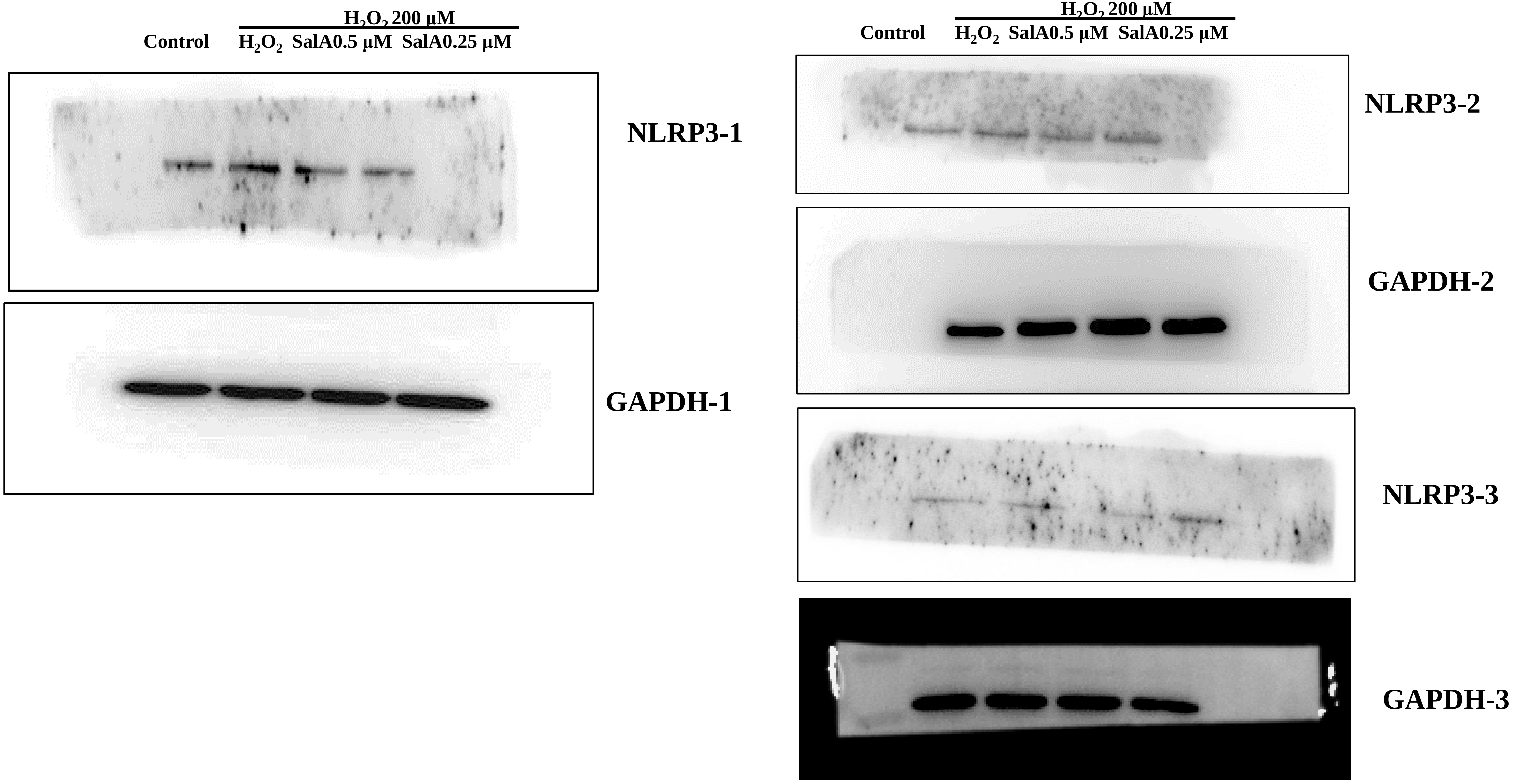
H2O2 200 μM
Control
H2O2
SalA0.5 μM
SalA0.25 μM
NLRP3-1
GAPDH-1
NLRP3-2
GAPDH-2
NLRP3-3
GAPDH-3

Figure 5 A

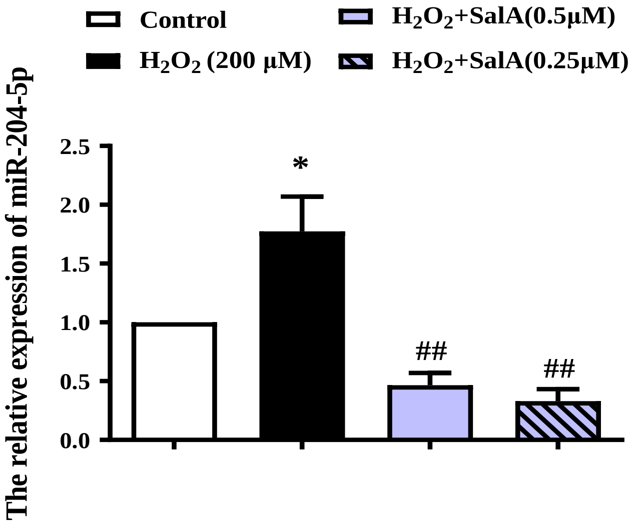

Figure 5A. The effect of SalA on the miR-204-5p expression of HUVECs induced by H_2O_2. (**P < 0.05* vs Control, $^{\#\#}$*P<0.01* vs H_2O_2).

B

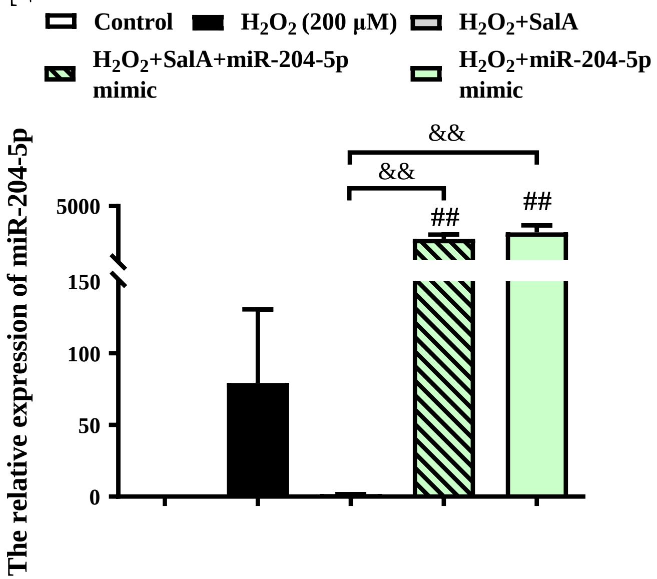

C

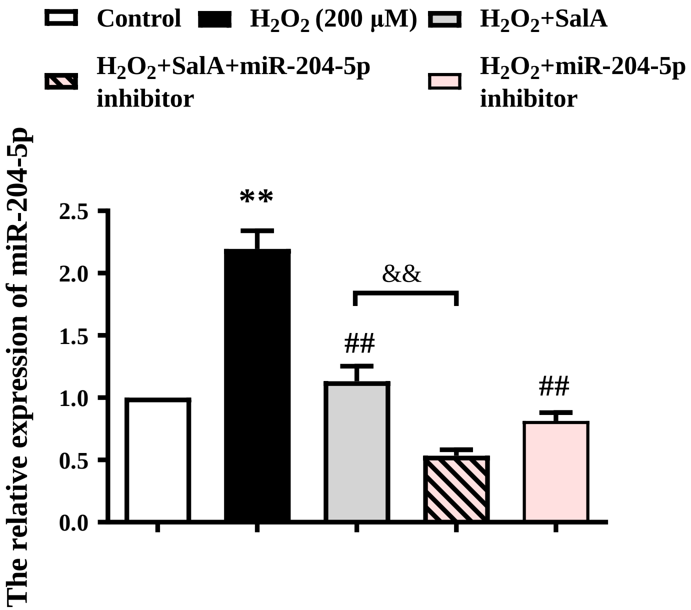

Figure 5B-5C. The effect of SalA on the miR-204-5p expression of H_2O_2-induced HUVECs with miR-204-5p mimic/inhibitor.
((**P<0.05,* ***P<0.01* vs Control, $^{\#\#}$*P<0.01* vs H_2O_2, $^{\&\&}$*P<0.01* vs H_2O_2 + SalA).

Figure 6

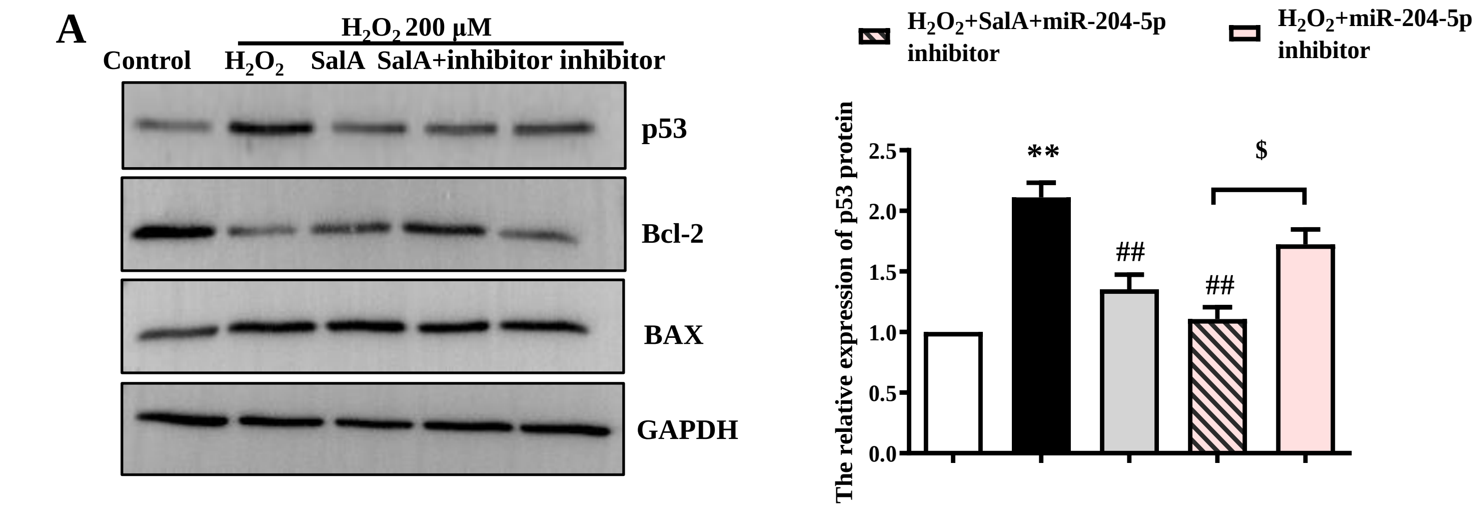

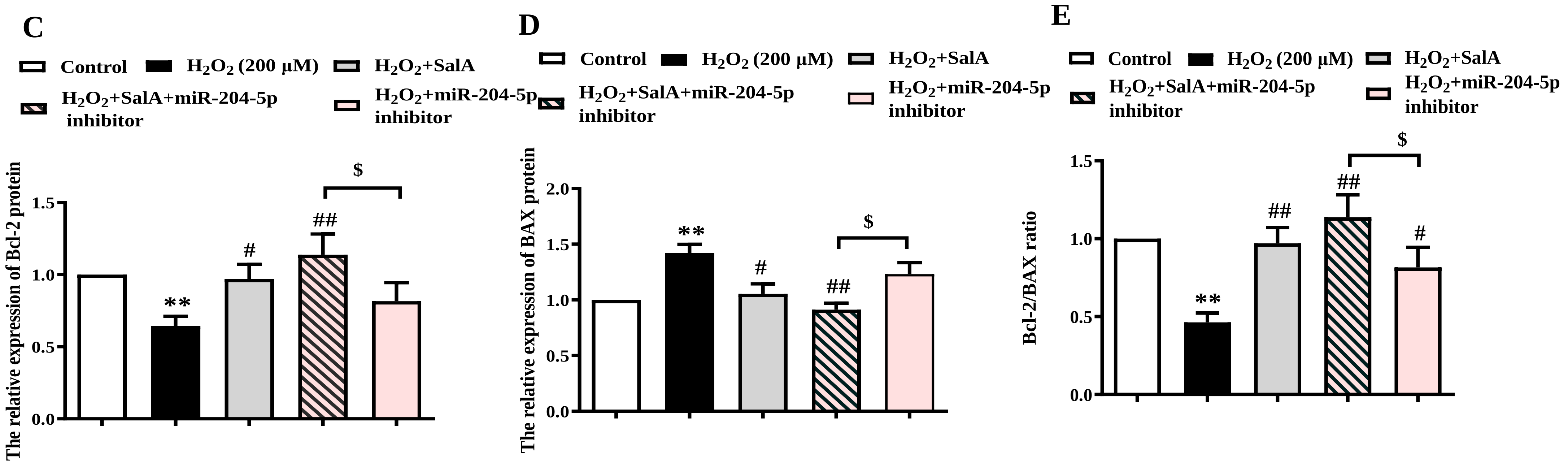

Figure 6. Effects of SalA on expression of apoptosis-related protein in H_2O_2-induced HUVECs by inhibiting miR-204-5p.

(A) The effect of SalA on the protein expression of (B) p53, (C) Bcl-2 and (D) BAX were detected by western blotting. (E)The expression ratio of Bcl-2/BAX.

(***P<0.01* vs Control, $^{\#}$*P<0.05*, $^{\#\#}$*P<0.01* vs H_2O_2, $^{\$}$*P<0.05* vs H_2O_2 + miR-204-5p inhibitor).

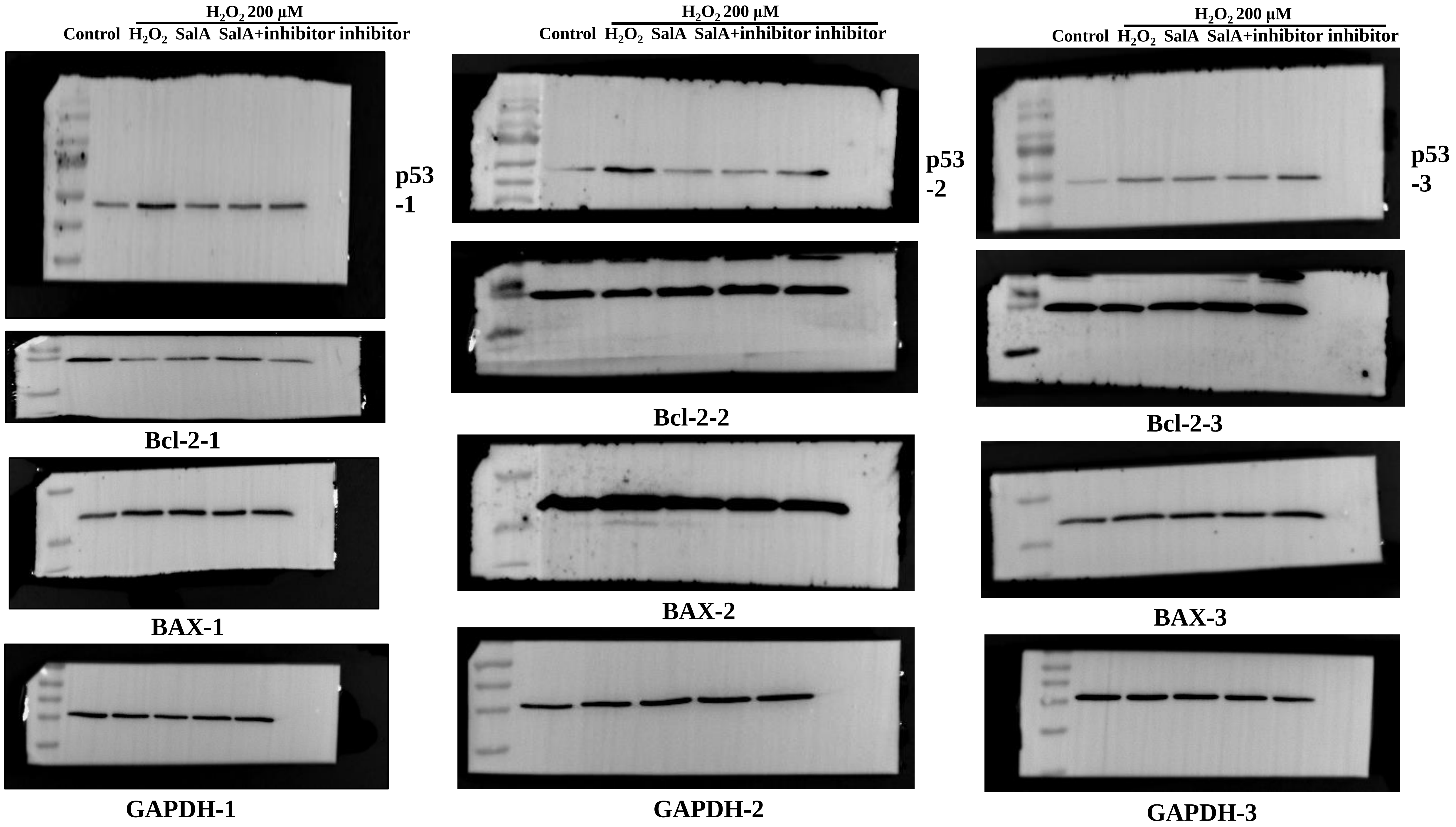

H2O2 200 μM
Control H2O2 SalA SalA+inhibitor inhibitor
p53-1
Bcl-2-1
BAX-1
GAPDH-1
p53-2
Bcl-2-2
BAX-2
GAPDH-2
p53-3
Bcl-2-3
BAX-3
GAPDH-3

Figure 7. SalA could alleviate H_2O_2-induced oxidative injury by downregulating miR-204-5p in HUVECs.

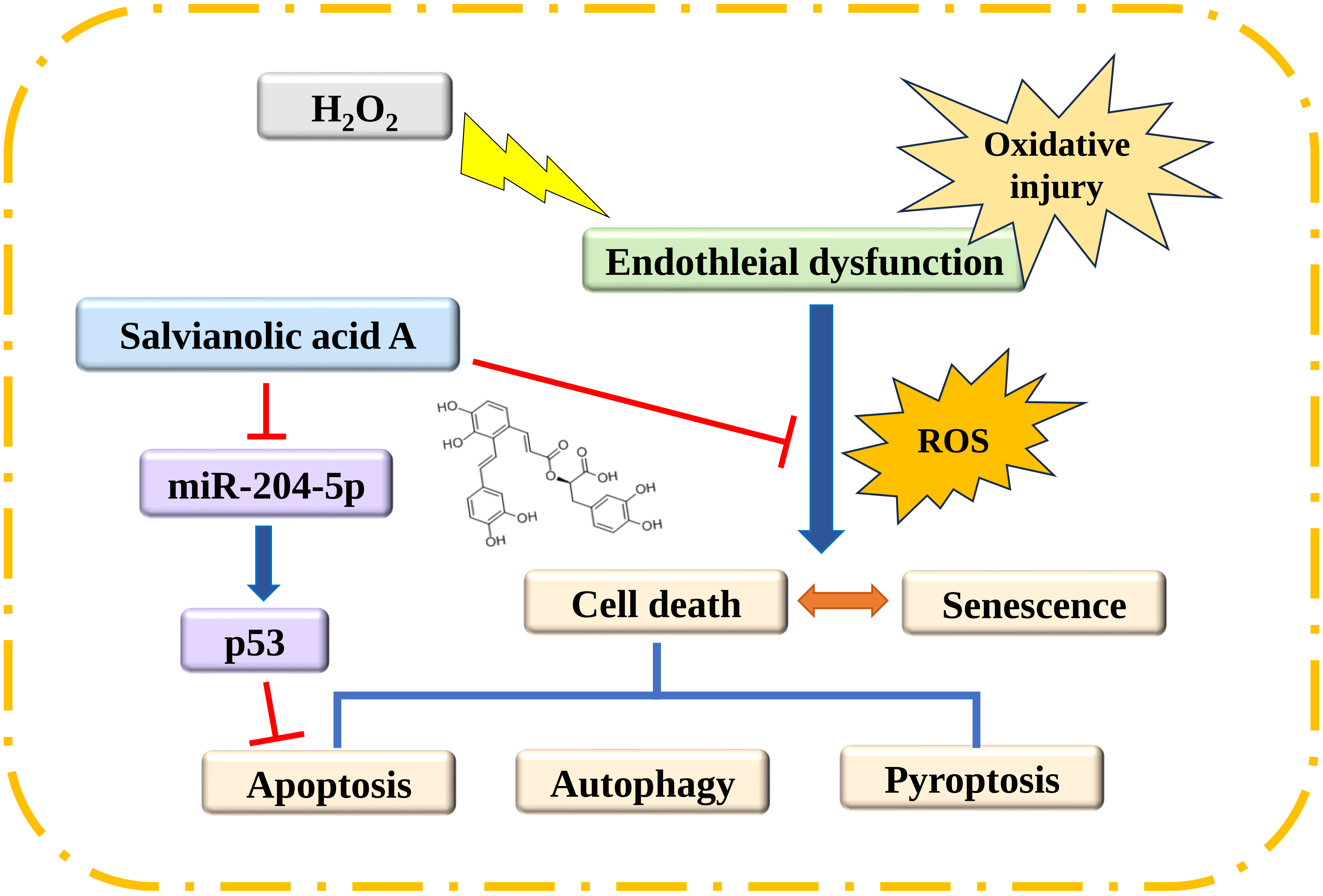

Supplement: Supplementary file 1 — Supplementary Information. [file 41598_2024_62556_MOESM1_ESM.pdf]
